# A systematic quality evaluation and review of nanomaterial genotoxicity studies: a regulatory perspective

**DOI:** 10.1186/s12989-022-00499-2

**Published:** 2022-09-14

**Authors:** Kirsi K. Siivola, Michael J. Burgum, Blanca Suárez-Merino, Martin J. D. Clift, Shareen H. Doak, Julia Catalán

**Affiliations:** 1 Finnish Institute of Occupational Health, Box 40, Työterveyslaitos, 00032 Helsinki, Finland; 2 In Vitro Toxicology Group, Faculty of Medicine, Health and Life Sciences, Institute of Life Sciences, Swansea University Medical School, Singleton Park, Swansea, SA2 8PP Wales UK; 3 TEMAS Solutions GmbH, 5212 Hausen, Switzerland; 4 Department of Anatomy Embryology and Genetics, University of Zaragoza, 50013 Zaragoza, Spain

**Keywords:** Genotoxicity, Mutagenicity, Nanoparticles, Nanofibers, Nanotubes, Quality, Reliability, Relevance, Databases, Assays

## Abstract

The number of publications in the field of nanogenotoxicology and the amount of genotoxicity data on nanomaterials (NMs) in several databases generated by European Union (EU) funded projects have increased during the last decade. In parallel, large research efforts have contributed to both our understanding of key physico-chemical (PC) parameters regarding NM characterization as well as the limitations of toxicological assays originally designed for soluble chemicals. Hence, it is becoming increasingly clear that not all of these data are reliable or relevant from the regulatory perspective. The aim of this systematic review is to investigate the extent of studies on genotoxicity of NMs that can be considered reliable and relevant by current standards and bring focus to what is needed for a study to be useful from the regulatory point of view. Due to the vast number of studies available, we chose to limit our search to two large groups, which have raised substantial interest in recent years: *nanofibers* (including nanotubes) and *metal-containing nanoparticles*. Focusing on peer-reviewed publications, we evaluated the completeness of PC characterization of the tested NMs, documentation of the model system, study design, and results according to the quality assessment approach developed in the EU FP-7 GUIDEnano project. Further, building on recently published recommendations for best practices in nanogenotoxicology research, we created a set of criteria that address assay-specific reliability and relevance for risk assessment purposes. Articles were then reviewed, the qualifying publications discussed, and the most common shortcomings in NM genotoxicity studies highlighted. Moreover, several EU projects under the FP7 and H2020 framework set the aim to collectively feed the information they produced into the eNanoMapper database. As a result, and over the years, the eNanoMapper database has been extended with data of various quality depending on the existing knowledge at the time of entry. These activities are highly relevant since negative results are often not published. Here, we have reviewed the NanoInformaTIX instance under the eNanoMapper database, which hosts data from nine EU initiatives. We evaluated the data quality and the feasibility of use of the data from a regulatory perspective for each experimental entry.

**Supplementary Information:**

The online version contains supplementary material available at 10.1186/s12989-022-00499-2.

## Background

Nanomaterials (NMs) are materials with at least one size dimension between 1 and 100 nm [1]. Due to the reduced size and the corresponding increased surface area, NMs have unique properties suitable for many industrial applications that, however, also raise concerns about their adverse health effects [2, 3]. Therefore, NMs—especially metals, metal oxides and nanofibers—have been extensively investigated for their safety [2, 4].

An important concern related to the safety of NMs is their capacity to produce genetic damage [3, 5]. Unrepaired genotoxic events can lead to permanent changes in the genetic material (i.e., mutations) that, if occurring in critical genes, might lead to cancer [6]. Therefore, every mutagen is considered potentially carcinogenic [3]. Due to the important consequences to human health, mutagenicity is a hazard endpoint required in all product regulations and, consequently, a key endpoint in most of the testing strategies developed for NMs [5, 7–10].

There has been a steady rise in the number of nanogenotoxicology research papers published in the twenty-first century [11]. In parallel, a vast amount of data relevant to NM toxicity have been produced in several initiatives (e.g., EU projects). Yet, for regulatory purposes, these data may only be retrieved if published, which is generally not the case for negative results. Unfortunately, manual retrieve and review of information from publications is time consuming, which hampers re-use of data. As an example, a recent Scientific Committee on Consumer Safety (SCCS) Opinion on NMs in cosmetics could not resolve a conclusion on the use of SiO_2_ due to the lack of data [12]; yet a quick search under the eNanoMapper database provided at least 200 entries related to toxicity of this substance. It is therefore not clear if stakeholders are not aware of databases, if database searching is too time-consuming or if the information provided in these databases is of no use from a regulatory perspective.

Concomitantly, large research efforts have contributed to both our understanding of key physico-chemical (PC) parameters regarding NM characterization as well as the limitations of toxicological assays originally designed for soluble chemicals [5, 13, 14]. As a consequence, it is becoming increasingly clear that not all generated information is reliable or relevant from the regulatory perspective; that is, that it can give evidence of the clarity and plausibility of the findings, and of the extent to which data and tests are appropriate for hazard characterization [15].

In order to make use of the existing toxicological data, quality evaluation methods and recommendations for best practices in nanotoxicology research have been developed, e.g., in the EU FP-7 GUIDEnano [5, 16] and H2020 caLIBRAte projects [17], and by the International Life Sciences Institute (ILSI) Health and Environmental Sciences Institute (HESI) Genetic Toxicology Technical Committee (GTTC) [13]. Based on these research approaches, one key aspect is currently missing: understanding of how the obtained information fits to a regulatory approach.

Therefore, the aim of this systematic review was to investigate the extent of studies and data on genotoxicity of NMs that can be considered reliable and relevant by current standards and bring focus to what is needed for a study to be useful from the regulatory point of view. Peer-reviewed publications were evaluated for the completeness of PC characterization of the tested NMs, and reliability of the studies according to the GUIDEnano quality assessment approach. In addition, the relevance of the studies was assessed by a set of assay-specific criteria that were created building on recently published recommendations for best practices in nanogenotoxicology research. In parallel, a similar evaluation of the genotoxicity data retrieved from the NanoInformaTIX instance was performed with a main focus on the regulatory needs regarding data quality. The qualifying publications are discussed, and future research needs, based on the most common shortcomings found, are proposed.

## Literature search

The searches for peer-reviewed research publications on the genotoxicity of NMs were conducted in PubMed and Scopus and limited between 2009 and 2019. We focused on the two large groups of NMs—nanofibers (including nanotubes), and metal-containing nanoparticles (NPs)—for which there is more available information on the genotoxic potential in the literature and databases as compared with other NMs. The searches were performed separately for each group of NMs. Within each material type, separate searches were done for in vitro and in vivo studies.

The publications were limited to validated assays, according to the inclusion criteria stated in Table 1, where the search terms used for nanofibers and metal-containing NPs are also described. The bacterial gene mutation assays were omitted as they are generally uninformative for NMs due to limited particle uptake [18]. The in vitro single-cell gel electrophoresis (comet) assay was not included due to the considerable variation in protocols in absence of guidelines and standardization, and the possibility of false positive results owing to NM interfering with DNA after lysis [19]. On the other hand, all routes of exposure were accepted for the in vivo studies. As few studies performed by inhalation were expected, other methods of administration through the respiratory route—(oro)pharyngeal aspiration or intratracheal instillation—were considered, as they have been reported to be reliable methods for assessing the pulmonary outcomes of NM exposure [20, 21].

**Table 1:** Inclusion and exclusion criteria, and search terms used in the literature review

**Inclusion criteria**
1. In vitro studies performed using validated assays:
• Mammalian cell micronucleus (MN) assay
• Chromosomal aberration (CA) assay
• Mammalian gene mutation assays: tests using hypoxanthine–guanine phosphoribosyltransferase (*Hprt*) or xanthine phosphoribosyltransferase (*Xprt*), and thymidine kinase (*Tk*) genes, including the mouse lymphoma assay (MLA)^a^
2. In vivo studies performed using validated assays:
• In vivo comet assay
• Mammalian erythrocyte MN assay
• Mammalian CA assay
• Gene mutation assays
3. In vivo studies performed by inhalation, oropharyngeal aspiration, intratracheal instillation, dermal, oral exposure, or any type of injection
**Exclusion criteria**
4. The words fibers, nanotubes, graphene, and therapy, were excluded from the search for in vivo studies of metal-containing NPs, as the search produced a considerable number of publications unrelated to the topic of this review
5. Full text was not available in English through conventional sources
**Search terms**
6. In vitro nanofibers: (nanotubes OR nanofibers OR nanofibres) AND (Hprt OR Xprt OR Tk OR micronucleus OR "chromosomal aberration*") AND (human OR mouse OR rat OR mammalian)
7. In vivo nanofibers: (nanofibers OR nanofibres OR nanotubes) AND genotox* AND (“in vivo” OR rats OR mice)
8. In vitro metal-containing NPs: nanoparticles AND (Hprt OR Xprt OR Tk OR micronucleus OR "chromosomal aberration*") AND (human OR mouse OR rat OR mammalian) NOT thioketal
9. In vivo metal-containing NPs: nanoparticles AND (comet OR micronucleus OR aberrations OR "gene mutation") AND (inhalation OR aspiration OR instillation OR dermal OR oral OR gavage OR injection) AND metal NOT (fibers OR fibres OR graphene OR nanotubes OR therapy)^b^

^a^As TK is an acronym of thioketal, it was added as an excluding term within the in vitro metal-containing NPs search. ^b^As the search was limited to nanoparticles, some studies on high aspect ratio metal-containing nanomaterials (e.g., nanorods or nanowires) may have not been caught

The suitability of the publications produced by each search was confirmed based on the abstracts. The search terms for in vitro studies produced a number of in vivo studies not covered by the in vivo search and vice versa. These studies were moved under the correct topic and included in the evaluation. The publications, which included both in vitro and in vivo work, were evaluated in both groups for the corresponding parts.

## Quality evaluation of peer-reviewed literature

The quality of the studies was evaluated according to the method developed in the EU FP-7 GUIDEnano project [16]. The approach builds upon previous initiatives, mainly on that of Card and Magnusson [22] as far as human toxicity studies. The GUIDEnano approach consists of two scores related to completeness of substance characterization (S) and reliability of the study (K). The criteria included in each score, which were published earlier by Fernández Cruz et al*.* [16], are reproduced in Additional file 1: Tables S1–S3. Due to the broad scope of the GUIDEnano approach, it needs to be complemented with additional criteria to evaluate the relevance of the studies for each given environmental and human toxicity endpoint.

### Evaluation based on the GUIDEnano quality approach

First, the S score was applied to filter out studies with incomplete NM characterization. The S score (Additional file 1: Table S1) addresses whether a minimum set of key PC properties of NMs, both as provided and measured in the exposure medium, have been assessed in the study under consideration. Out of the selected parameters, some are considered as compulsory properties (‘red questions’, e.g., chemical composition, size, shape and surface chemistry), which disregard the study if they are not fulfilled [16]. In addition, studies are also disregarded if a minimum score is not reached.

For the studies that contained test results for more than one NM, the S score was determined separately for each NM and the NMs with acceptable material characterization were subjected to further evaluation.

Secondly, the K score was employed to assess the reliability of the studies with acceptable NM characterization. The K score (Additional file 1: Tables S2 and S3) was designed to ascertain an adequate description of the model system and study design, proper results documentation, and plausibility of the results. Different sets of questions are addressed to in vitro and in vivo studies, and some of them are identified as ‘red questions’. After being evaluated, the study is assigned to one of the categories established by Klimisch et al*.* [23]: K1 (reliable without restrictions), K2 (reliable with restrictions) or K3 (unreliable). Studies that failed in the latter category, either because they did not pass all the red questions or failed to reach the minimum score, were not considered for further assessment.

### Assay-specific evaluation of the genotoxicity assays

In addition to applying the GUIDEnano quality evaluation approach, we further assessed the relevance and limitations of genotoxicity assays used in testing NMs by creating a set of criteria that addresses the assay-specific details. The criteria were based on the recommendations set by Doak et al*.* [24], Pfuhler et al*.* [25], Elespuru et al*.* [13], Catalán et al*.* [5], and the corresponding OECD test guidelines (TGs) [26–33].

The criteria for the evaluation of the in vitro mammalian cell genotoxicity tests are presented in Table 2. Some criteria were considered as obligatory either because they are already required in the corresponding OECD TGs and considered, as such, applicable for NMs, or because there is strong enough evidence of their relevance for assessing the genotoxicity of NMs. Among the former e.g., background frequencies must be reported because excessively high levels may indicate an inappropriate experimental set-up, the positive controls should produce a statistically significant increase, or a concurrent cytotoxicity assessment using a validated parameter should be performed. The latter includes requesting that Cytochalasin B (Cyt-B)—an actin polymerization inhibitor commonly used to inhibit cytokinesis and enable identification of dividing cells [34]—should be added no less than 6 h after starting the exposure when performing the cytokinesis-block micronucleus (MN) assay. In this way, it is possible to ensure a period of exposure of the cells to the NM in the absence of Cyt-B, which may block the cellular uptake of the NMs [35, 36]. However, as this criterion was established to avoid including false negative results, clearly positive results could be accepted regardless of it.

**Table 2:** Criteria for the assay-specific evaluation of the validated in vitro mammalian cell genotoxicity assays. Obligatory criteria are presented in italics. The non-obligatory criteria were applied to evaluate the significance of the acceptable test results

**All in vitro assays**
1. Adequate dose range: Doses with no or low cytotoxicity and moderate cytotoxicity should be included. For non-toxic materials a maximum dose of ~ 250 µg/ml is considered adequate
2. If the result is positive only at highly cytotoxic doses (close to 50–60% cytotoxicity for micronucleus test or 10–20% survival for gene mutation tests) the conclusions of the study must be reevaluated
3. Cellular uptake should be confirmed
**Mammalian cell micronucleus (MN) test**
4. *Background MN frequency must be provided*
5. *The concurrent positive controls must elicit a statistically significant increase in MN frequency*
6. *Concurrent cytotoxicity assessment must be performed as described in OECD TG487 (with Cyt-B CBPI or RI; without Cyt-B RPD or RICC)*
7. *A minimum of 2000 cells have been scored per concentration. A minimum of two replicates or independent experiments have been performed*
8. *If Cytochalasin B is used, it is added ≥ 6 h after starting the exposure to allow uninhibited cellular uptake. Tests with positive results are accepted regardless of this criterion*
9. *There are no major problems in the study design (e.g. less than 3 doses with up to 50–60% cytotoxicity, only cytotoxic doses tested, harvesting schedule does not fall within 1.5–2 cell cycles, treatment time less than 3 h, inappropriate solvent without vehicle control, too high solvent concentration)*
10. *Treatment schedule: The cells have completed at least one cell cycle in the presence of the NM so that NMs taken up by the cells may come into direct contact with the DNA when the nuclear membrane breaks down during mitosis. Tests with positive results are accepted regardless of this criterion*
**Mammalian chromosomal aberration (CA) test**
11. *Background CA level must be provided*
12. *Positive control induces a statistically significant increase in CAs*
13. *Concurrent cytotoxicity assessment is performed as described by the OECD guideline (RPD or RICC, MI is acceptable for primary lymphocytes)*
14. *A minimum of 300 metaphases per concentration (unless clearly positive response) are analyzed. A minimum of two replicates or independent experiments are performed*
15. *There are no major problems in the study design (e.g., less than 3 doses with up to 50–60% cytotoxicity, only cytotoxic doses tested, clearly inadequate treatment time, harvesting schedule does not fall within 1.5 cell cycles, inappropriate solvent without vehicle control, too high solvent concentration)*
16. *Treatment schedule: The cells have completed at least one cell cycle in the presence of the NM so that NMs taken up by the cells may come into direct contact with the DNA when the nuclear membrane breaks down during mitosis. Tests with positive results are accepted regardless of this criterion*
**Mammalian cell *Hprt*, *Xprt*, and *Tk* gene mutation tests and mouse lymphoma assay (MLA)**
17. *Negative control mutation frequency must be reported*
18. *The concurrent positive controls must elicit a statistically significant increase in mutant frequency*
19. *Concurrent cytotoxicity evaluation must be performed with appropriate cytotoxicity parameter, as described by OECD TGs (RS for Hprt/Xprt/Tk; RTG for MLA)*
20. *A minimum of two replicates or independent experiments are performed*
21. *There are no major problems in the study design (e.g., less than 4 doses above 10–20% survival, only cytotoxic doses tested, treatment time less than 3 h, inadequate phenotypic expression time—for MLA 2 days, for Tk 3–4 days, for Hprt/Xprt a minimum of 7–9 days, inappropriate solvent without vehicle control, too high solvent concentration)*
22. *Treatment schedule: The cells have completed at least one cell cycle in the presence of the NM so that NMs taken up by the cells may come into direct contact with the DNA when the nuclear membrane breaks down during mitosis. Tests with positive results are accepted regardless of this criterion*

Another obligatory criterion refers to the treatment schedule. In order to be able to induce primary direct genotoxicity, NMs should be internalized by cells and come in contact with the genetic material [3]. As some NMs cannot pass through the nuclear membrane, optimally the cells should complete at least one cell cycle in the presence of the NM so that internalized NMs may come into direct contact with the DNA when the nuclear membrane breaks down during mitosis [37, 38]. However, again both requirements concern mainly negative results, whereas clearly positive results can be considered relevant regardless of proof of cellular uptake or adequate treatment time. It should be noted that regarding treatment times the current OECD guidelines for in vitro genotoxicity testing may not be suitable for NMs. Adaptation and validation of OECD guidelines for testing NMs is currently in progress under the umbrella of the OECD Working Party on Manufactured Nanomaterials, e.g., project 4.95: Guidance Document on the Adaptation of In Vitro Mammalian Cell Based Genotoxicity TGs for Testing of Manufactured Nanomaterials [39].

We also established a set of non-obligatory criteria for the purpose of collecting information that could be used for assessing the assay relevance on a case-by-case basis. These criteria were not considered compulsory as they are not required in the guidelines or are more difficult to apply to the NMs. For instance, the highest test concentration of 2 mg/ml established by the OECD TGs for low toxicity substances (not reaching the 55 ± 5% cytotoxicity limit) is generally recognized as excessive for NMs. High concentrations that lead to extensive agglomeration should be avoided [40]. Furthermore, artefactual positive effects could be induced by suffocation of the cells as a consequence of the high amount of deposited material [41]. However, lower top dose limits of 100 µg/ml [40] or 200 µg/ml (EU NANoREG project) have only been recently suggested and would also depend on the dispersibility and stability of each specific material. Hence, we decided to set a 250 µg/ml limit that could fit to most of them.

The evaluation criteria for the mammalian in vivo genotoxicity assays are listed in Table 3. As before, obligatory, and non-obligatory criteria were established. Among the former, the inclusion of an appropriate cytotoxicity parameter or including a sufficient number of cells to reach acceptable sensitivity, according to the last version of the corresponding OECD guideline [30–32], were required. However, in case of clearly positive results and a negative control within a normal range in the MN and chromosomal aberration (CA) tests, also adherence to a previous version of the same guidelines (OECD TG 473 and 474, respectively) [42, 43] was considered acceptable.

**Table 3:** Criteria for the assay-specific evaluation of the validated in vivo genotoxicity assays. Obligatory criteria are presented in italics. The non-obligatory criteria were applied to evaluate the significance of the acceptable test results

**All in vivo assays**
1. *Positive and negative control data must be available*
2. An adequate dose range should include at least three doses covering a range from the maximum tolerated dose, 2000 mg/kg body weight for < 14-day or 1000 mg/kg body weight for > 14-day exposures in oral exposure, or lung overload limit (threshold level of particles reached within the lung above which the observed adverse effects may be attributable to particle accumulation and may not reflect a real toxic response) for inhalation exposure, to a dose producing little or no toxicity. The maximum dose may also be defined by toxicity in the target tissue or by a particle concentration which, in a real-life exposure scenario, becomes effectively non-nano due to particle agglomeration
3. The exposure route should be justified by human exposure
4. Tissue distribution data should be provided. It should be demonstrated that the material itself, its metabolites or secondary effectors reach the target tissues
**Mammalian erythrocyte micronucleus (MN) test**
5. *The proportion of reticulocytes (immature erythrocytes, RET%) among total erythrocytes must be determined for each animal by counting a total of at least 500 erythrocytes for bone marrow and 2000 erythrocytes for peripheral blood*
6. *At least 4000 immature erythrocytes per animal should be scored for the incidence of micronucleated immature erythrocytes and a minimum of 5 animals per group should be analyzed to reach sufficient statistical power*
7. *When peripheral blood is used, it must be established that splenic removal of micronucleated cells from the circulation does not compromise the detection of induced NM in the species selected (this has been clearly demonstrated for mouse and rat peripheral blood)*
8. *Sample collection times at which the treatment-related induction of micronucleated immature erythrocytes can be detected are required. In the case of peripheral blood sampling, enough time must also have elapsed for these events to appear in circulating blood. The time required for the nanomaterial to reach the target tissue should be considered in the experimental design. Treatment schedule should be scientifically justified, e.g., with biodistribution data, or by minimum, following the OECD TG. Tests with positive results should be accepted regardless of this criterion*
**Mammalian bone marrow chromosomal aberration (CA) test**
9. *The mitotic index must be determined as a measure of cytotoxicity in at least 1000 cells per animal for all treated animals*
10. *At least 200 metaphases per animal (or more if negative control frequency is < 1%) and a minimum of 5 animals per group must be analyzed to reach sufficient statistical power*
11. *Treatment schedule should be scientifically justified, e.g., with the help of biodistribution data, or by minimum following the OECD TG. For rodents, the first sampling interval should be the time necessary to complete 1.5 normal cell cycle lengths after the exposure of the target tissue. The time required for the nanomaterial to reach the target tissue as well as its effect on cell cycle kinetics can affect the optimum time for CA detection and should be considered in the experimental design. A later sample collection 24 h after the first sampling time is recommended for the highest dose. Tests with positive results should be accepted regardless of this criterion*
**Mammalian alkaline comet assay**
12. *Automated or semi-automated (quantitative) scoring required, % tail DNA from at least 150 cells per sample (excluding hedgehogs) and a minimum of 5 animals per group are required*
13. *For positive results, an examination of one or more indicators of cytotoxicity (e.g. histopathology or trypan blue exclusion) is required. Increases in DNA migration in the presence of clear evidence of cytotoxicity should be interpreted with caution*
14. *According to the OECD TG, the samples should be collected no later than 6 h after the final treatment. However, the time required for the nanomaterial to reach the target tissue and biopersistence of the materials should be considered, and positive results accepted with any schedule. For negative results the sample collection schedule should be justified preferably by biodistribution data, and it should be considered that the OECD TG recommends two or more treatments at approximately 24 h intervals*
**Erythrocyte Pig-a gene mutation assay**
15. *A sufficient number of cells to detect at least one mutant cell per sample and a minimum of six animals per group must be analyzed*
16. *For optimal sensitivity, a 28-day repeated exposure is recommended, and samples should be analyzed at least once within a few days after cessation of exposure (days 29–31 after the first dose). Other treatment schedules should be scientifically justified. Tests with positive results should be accepted regardless of this criterion*

The most critical issue related to the in vivo assessment of NMs refers to the treatment schedule to ensure that enough amount of the material has reached the target tissue and exerted an effect at the time of sample collection [5, 25]. The treatment schedule and sampling times were evaluated for all the in vivo assays, and tests with inadequate schedules were assessed case-by-case considering the results and the available biodistribution data. Although the correct time window for sampling in different exposure scenarios is described in the OECD guidelines, it may take longer for some NMs to accumulate in target tissue and thus the optimal time window could be later than described in the guideline [5, 24, 25]. However, negative results from samples collected several days or months later than recommended are not meaningful without biodistribution data showing that the material has, in fact, accumulated, and is biopersistent. Positive results were accepted regardless of an unjustified treatment schedule.

As a non-compulsory criterion, the availability of biodistribution data was evaluated to establish the extent in which the NM has reached the target tissue measured in the genotoxicity assay. This is particularly important when evaluating the relevance of negative test results. Despite its relevance, the criterion was considered as non-obligatory as a new TG (or an amendment to the existing TG 417 on Toxicokinetics) to accommodate to NMs is currently in progress [39].

## Literature quality evaluation results

### Overview of publications relevant for regulatory risk assessment

About 52% (246) and 38% (58) of the original 468 and 152 literature search results for metal-containing NPs and nanofibers, respectively, were excluded because either the study was not in the scope of this review, the full-text access was not available, or results were already covered by the other search terms. The substance (S) score was finally evaluated in 137 in vitro studies and 85 in vivo studies on metal-containing NPs, and in 57 in vitro studies and 37 in vivo studies on nanofibers, which fitted the scope of the present review.

**Fig. 1:**
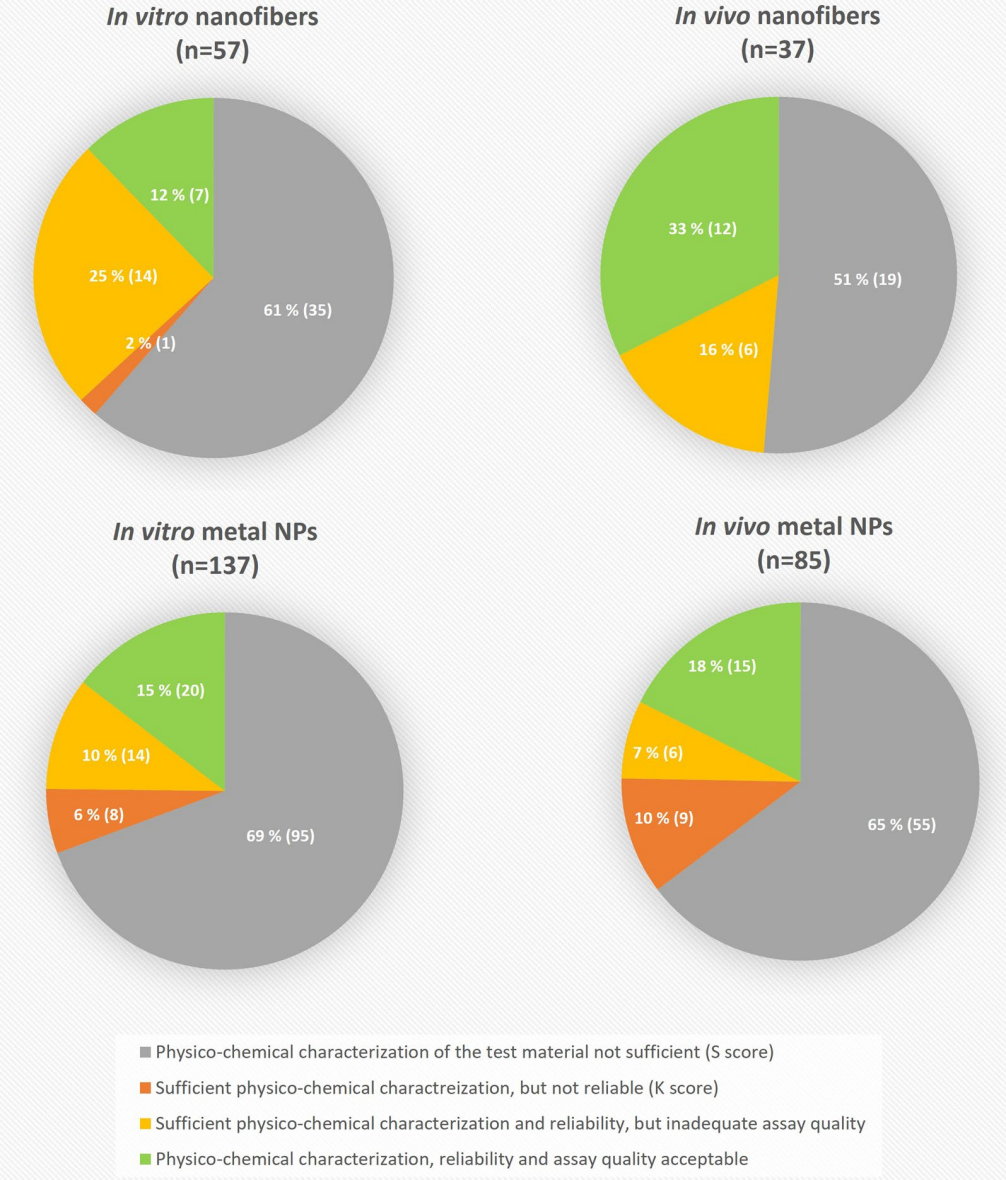
Quality assessment of genotoxicity studies on metal-containing nanoparticles and nanofibers. The percentage and total number (in parenthesis) of studies evaluated in each step are presented

The quantity of studies on nanofibers or metal-containing NPs evaluated in each step of the quality assessment is displayed in Fig. 1. Within all groups, by far the largest number of studies were rejected due to an incomplete PC characterization (S score) of the tested NM. For metal-containing NPs, a slightly larger proportion of studies (69% and 65% for i*n vitro* and in vivo, respectively) were rejected on this basis compared to nanofibers (61% and 51%, respectively). According to the GUIDEnano reliability evaluation (K score), 6% and 10% of the in vitro and in vivo studies on metal-containing NPs, respectively, were deemed unreliable, whereas all except one (2%) nanofiber studies were considered reliable. The differences may be due to the considerably smaller number of publications on nanofibers, but as the evaluations for each type of NM were conducted by a different evaluator, subjective interpretation of the evaluation criteria cannot be entirely ruled out. The proportion of in vitro studies, which passed all stages of the evaluation was similar for both metal-containing NPs and nanofibers, 15% and 12%, respectively. In case of the in vivo studies, 18% of the metal-containing NPs and 33% of the nanofiber publications were finally accepted.

### Results of the quality evaluation based on the GUIDEnano quality approach

The S score was the most limiting of the evaluation steps (Fig. 1). Acceptable NM characterization was only available in 31% and 39% of the in vitro studies, and 35% and 49% of the in vivo studies, for metal-containing NPs and nanofibers, respectively. The information was available either in the publications themselves or other clearly indicated sources. Failing to fulfill the acceptable substance characterization mostly stemmed from failing at least one of the ‘red questions’ (obligatory criteria), and less frequently from failing to fulfill the minimum points required in the scoring. Up to 65% and 28% of the in vitro studies, and 58% and 29% of the in vivo studies, for metal-containing NPs and nanofibers, respectively, failed at least one of the red questions. This is in line with the findings by Fernández-Cruz et al*.* [16], who found insufficient characterization of the tested NMs to be the principal weakness in the toxicity studies evaluated during the development of the GUIDEnano approach.

The most common shortcomings in the substance characterization were failures to provide the purity of the NM and the size distribution during the exposure, which is also in agreement with the GUIDEnano’s evaluation [16]. In many papers which had an acceptable S score in this evaluation, the purity of a well-identified commercial NM could be found from the supplier's website. This was deemed acceptable in the evaluation, although it should be noted that information on websites often becomes unavailable over time, and quite detailed information (e.g., a product and a lot number) is often required to identify the exact product from the supplier's website. Other reasons for failing this question included previously published characterization data that was either mis-referenced or the previous publication was not available from typical sources. Purity with respect to the nanofibers was a much more obtainable criterion compared with metal-containing NPs. The characterization of carbon nanotubes (CNTs) is fundamentally linked to their carbon purity and is an extremely common feature of their published PC feature set. The question of purity becomes especially pertinent when discriminating e.g*.*, between CNTs NM400 and NM401 which are extremely alike with the exception of higher metal impurity content in the NM400, an artifact of the catalysis. Nevertheless, it is highly recommended to provide a summary of the material characteristics provided by previous publications, the substance supplier’s website, or the certificate of analysis in the same publication with the genotoxicity test results.

As concerns the assessment of the size distribution during the exposure, dynamic light scattering (DLS) is one of the most used methods for characterizing NPs in the exposure medium. However, it may give an inaccurate size distribution for non-spherical materials. Transmission electron microscopy (TEM) is a more suitable method for accurate measurements of the particle size for nanofibers, although it does not provide a good estimation of the size distribution in the sample. In some conditions, particle size in medium changes with concentration [44]. Although measuring particle size from exposure medium in all the tested doses is not required by the S score nor used as a criterion in our evaluation, a dose-dependent particle size may contribute to a better interpretation of results.

Another red question with which we encountered some shortcomings concerned the shape of the primary particle, which was not always well described for the metal-containing NPs. Ideally this information should be clearly given, also for spherical or amorphous NMs. In this evaluation, however, we accepted TEM images as proof of particle shape.

After evaluating the completeness of material characterization, a total of 64 in vitro studies (42 and 22 metal-containing NPs and nanofibres publications, respectively) and 48 in vivo studies (30 and 18 metal-containing NPs and nanofibres publications, respectively) were further assessed for the quality.

The reliability of the studies was evaluated by the K score (Fig. 1). For nanofibers, only one in vitro study was considered unreliable due to failing at least one of the essential criteria. In the case of metal-containing NPs, most of the studies with a complete material characterization—81% in vitro and 74% in vivo—were also considered reliable. Failing at least one of the ‘red questions’ was the reason for not succeeding in passing the K score in all the cases. In the in vitro studies, the most common shortcomings were failures to clearly describe the source of the test system, and the study endpoints and methods. Among the in vivo studies, the most critical shortcoming was the lack of positive controls. Our findings comfort with those of Fernández-Cruz et al*.* [16] who concluded that, in general, peer-reviewed publications complied with the majority of the questions included in the K score. Nevertheless, and as mentioned before for material characterization, also for the description of methods it is generally not advisable to refer to sources that may change or become unavailable over time. Instead, all relevant details should be cited in the same publication with the genotoxicity data to ensure accessibility.

### Results of the assay-specific evaluation

A total of 55 in vitro studies (34 and 21 metal-containing NPs and nanofibres publications, respectively) and 39 in vivo studies (21 and 18 metal-containing NPs and nanofibres publications, respectively) were considered reliable in the K score-based evaluation. These studies were further assessed according to the assay-specific criteria detailed in Tables 2 and 3.

#### In vitro assays

**Fig. 2:**
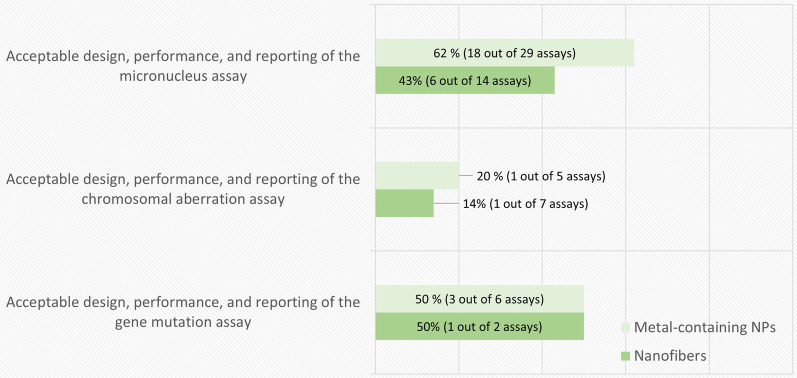
Results of the in vitro genotoxicity assay-specific evaluation. The percentage and number of assays (out of the total number of each specific type of assay) that fulfilled the assay-specific criteria is presented in each bar

From the total of 55 in vitro publications, the number of reported MN, CA and gene mutation assays were 29, 5 and 6 for metal-containing NPs, and 14, 7 and 2 for nanofibers, respectively. In some of the publications, more than one type of assay was used. Figure 2 summarizes the proportion of studies that successfully fulfilled the assay-specific criteria listed in Table 2. About half of the studies performed with the MN and gene mutations assays complied with the criteria. However, the proportion was much lower when using the CA assay.

**Fig. 3:**
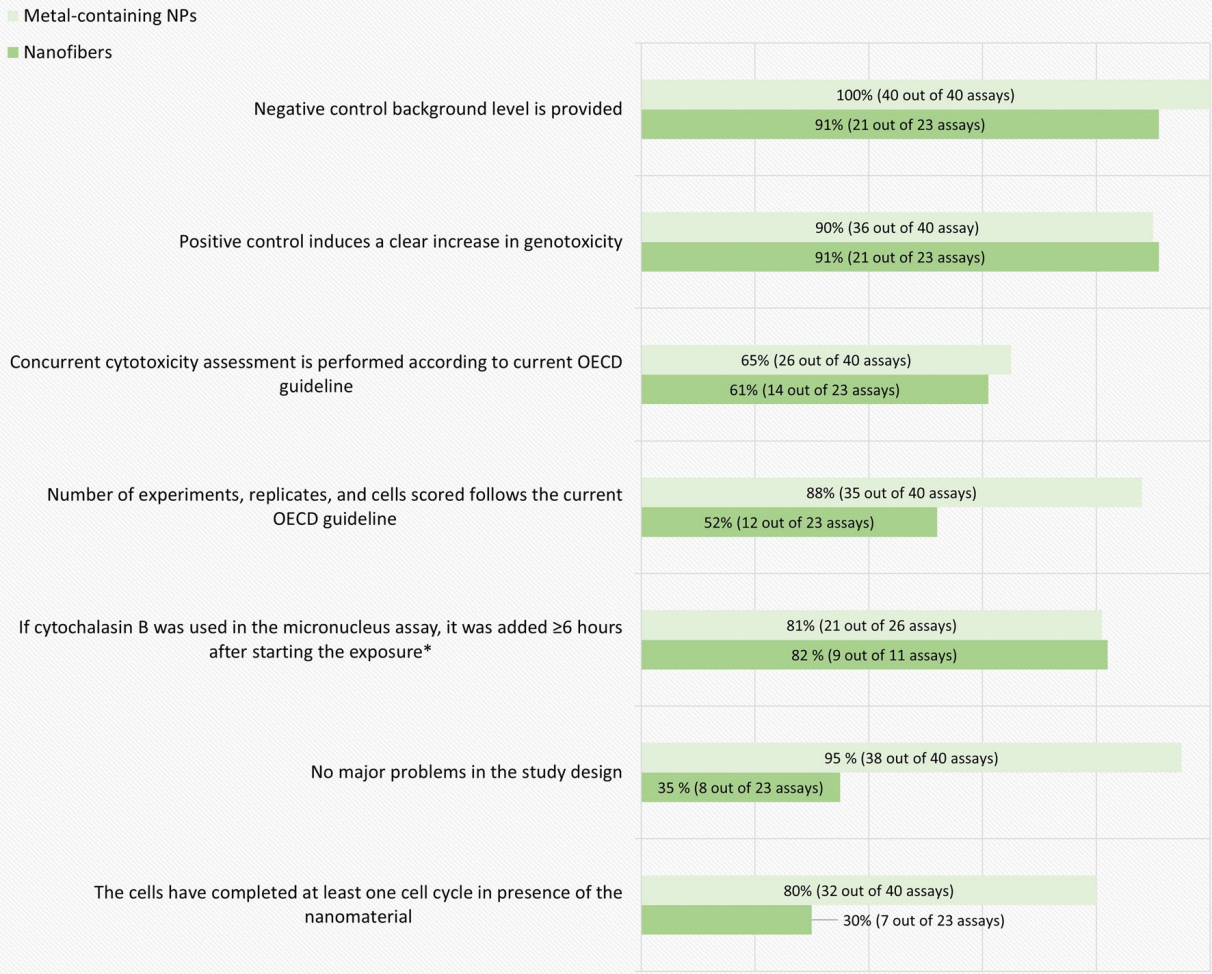
The breakdown of the in vitro genotoxicity assay-specific evaluation results by obligatory criteria. The percentage and number of assays (out of the total number of assays evaluated for each criterion) that fulfilled each assay-specific criterion is presented in each bar. *Evaluated only in the assays where Cytochalasin B was used. Clearly positive test results were considered acceptable regardless of this criterion

A breakdown of the in vitro assay quality evaluation results according to the obligatory criteria is shown in Fig. 3. The most common shortcoming for all types of materials leading to exclusion among in vitro genotoxicity tests was the lacking or inadequate concurrent cytotoxicity measurement. In addition, studies on nanofibers had a markedly larger number of shortcomings concerning the treatment schedule, study design and adherence to sample sizes or number of replicates or experiments recommended by the OECD guidelines compared to studies on metal-containing NPs.

The purpose of a concurrent cytotoxicity measurement is to ascertain a relevant dose range for the genotoxicity analysis. According to the OECD guidelines, a minimum of three (MN and CA assays), or four (gene mutation tests) doses should be tested up to a highest test concentration aiming at 50–60% cytotoxicity in case of MN and CA assays, and 10–20% survival in case of the gene mutation tests (criteria 9, 15 and 21 of Table 2). Genotoxicity analysis at excessively toxic concentrations could lead to false positive outcomes [5, 24]. On the other hand, testing only doses with too low toxicity may prevent or underestimate the detection of the genotoxic potential of the NMs [5, 45, 46]. A concurrent cytotoxicity assessment using a recommended cytotoxicity parameter based on cell proliferation is required (as specified in the criteria 6, 13 and 19 of Table 2). Assessing cytotoxicity in the same experiment as the genotoxicity predictors is especially important when testing NMs as some of them exhibit poor repeatability between dispersions.

The treatment schedule could be considered clearly inadequate for detection of direct genotoxicity in 20% and up to 70% of the evaluated genotoxicity tests for metal-containing NPs and nanofibers, respectively (Fig. 3). A much larger proportion of the studies did not describe the exposure time relative to the cell cycle or include any information about the length of the cell cycle in the chosen model system. In this evaluation the treatment time was considered acceptable if it was in the range of normal average cell cycle length (15–24 h). The test result, however, could also be considered acceptable by expert judgement regardless of the inadequate treatment time in case of clearly positive results.

**Fig. 4:**
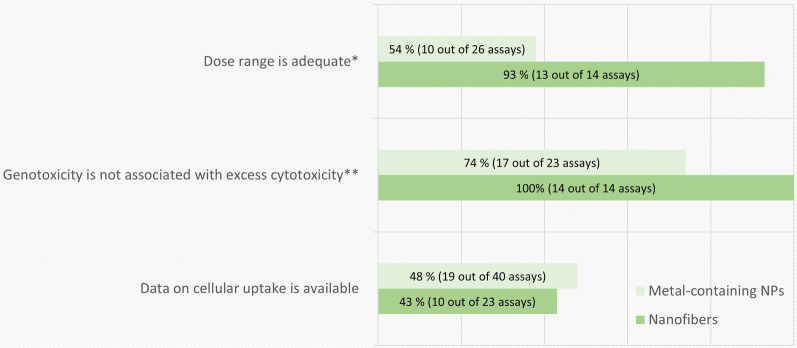
The breakdown of the in vitro genotoxicity assay-specific evaluation results by non-obligatory criteria. The percentage and number of assays (out of the total number of assays evaluated for each criterion) that fulfilled each assay-specific criterion is presented in each bar. *Evaluated in 26 and 14 assays (metal-containing NPs and nanofibers, respectively) for which a concurrent cytotoxicity assessment was performed. **Evaluated in 23 and 14 assays (metal-containing NPs and nanofibers, respectively) with a concurrent cytotoxicity assessment and positive results

The breakdown of the in vitro genotoxicity assay quality evaluation results according to the non-obligatory criteria is shown in Fig. 4. The main shortcoming for these criteria was the lack of confirmation of cellular uptake. As mentioned above, in order to come in contact with the genetic material, NMs should be internalized by cells. In this evaluation we did not consider assessment of particle uptake a mandatory requirement but used it as supporting information for the purpose of compensating other shortcomings and weighing the relevance of negative test results. An example of a well performed investigation of cellular uptake in the test system is the study by Di Bucchianico et al*.* [47]. These authors tested the time and dose dependence of Ni and NiO NPs uptake by inductively coupled plasma mass spectrometry (ICP-MS) and confirmed the presence of Ni in particle form at the last analyzed time point by TEM. They found that the uptake was rapid and dose dependent in the chosen test system. However, the same cannot be assumed for other cell lines, NMs, or methods of dispersion, and thus, especially in the case of negative genotoxicity test results, it is important that the study describes the capability of the cell line to internalize the material at the same time points and in the same conditions in which the genotoxicity test was performed.

Almost half of the assays for metal-containing NPs that included a concurrent cytotoxicity measurement failed to choose an appropriate dose range. In addition, in one fourth of the assays showing a positive outcome, the positive result was associated to excessive toxic doses (Fig. 4). As explained above, covering an appropriate dose range is critical for a proper interpretation of the genotoxicity outcomes. Some of the evaluated studies did only analyze non-toxic or very low cytotoxicity doses. On the other hand, others did not include enough low toxicity doses, which may have enabled a more reliable interpretation of the positive test results. We acknowledge fulfilling the regulatory requirements for an acceptable dose range with NMs may be challenging due to methodological limitations. At high doses heavy agglomeration of the test material may occur, making the test material effectively non-nano, or microscopical analysis can be hindered by material agglomerates covering the cell surface. However, in this case a clear description of the limitations and justification for the tested dose range should be given.

#### In vivo assays

**Fig. 5:**
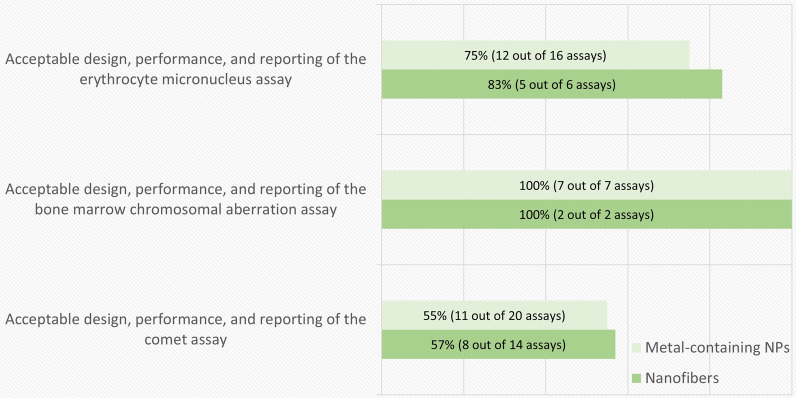
Results of the in vivo genotoxicity assay-specific evaluation. The percentage and number of assays (out of the total number of each specific type of assay) that fulfilled the assay-specific criteria is presented in each bar. Results from the erythrocyte Pig-a gene mutation assay are not shown in the figure (0 out of total 3 tests were acceptable for metal-containing nanoparticles (NPs), whereas no tests for nanofibers were retrieved in the literature search)

From the total of 39 in vivo publications, the number of reported MN, CA, comet and gene mutation (Pig-a) assays were 16, 7, 20 and 3 for metal-containing NPs, and 6, 2, 14 and 0 for nanofibers, respectively. In most of the publications, more than one type of assay was used. Figure 5 summarizes the proportion of studies that successfully fulfilled the assay-specific criteria listed in Table 3. All the studies performed with the CA assay, as well as most of those using the MN assay, complied with the criteria. However, almost half of the studies using the comet assay did not. On the other hand, none of the studies involving the erythrocyte Pig-a gene mutation assay were acceptable.

**Fig. 6:**
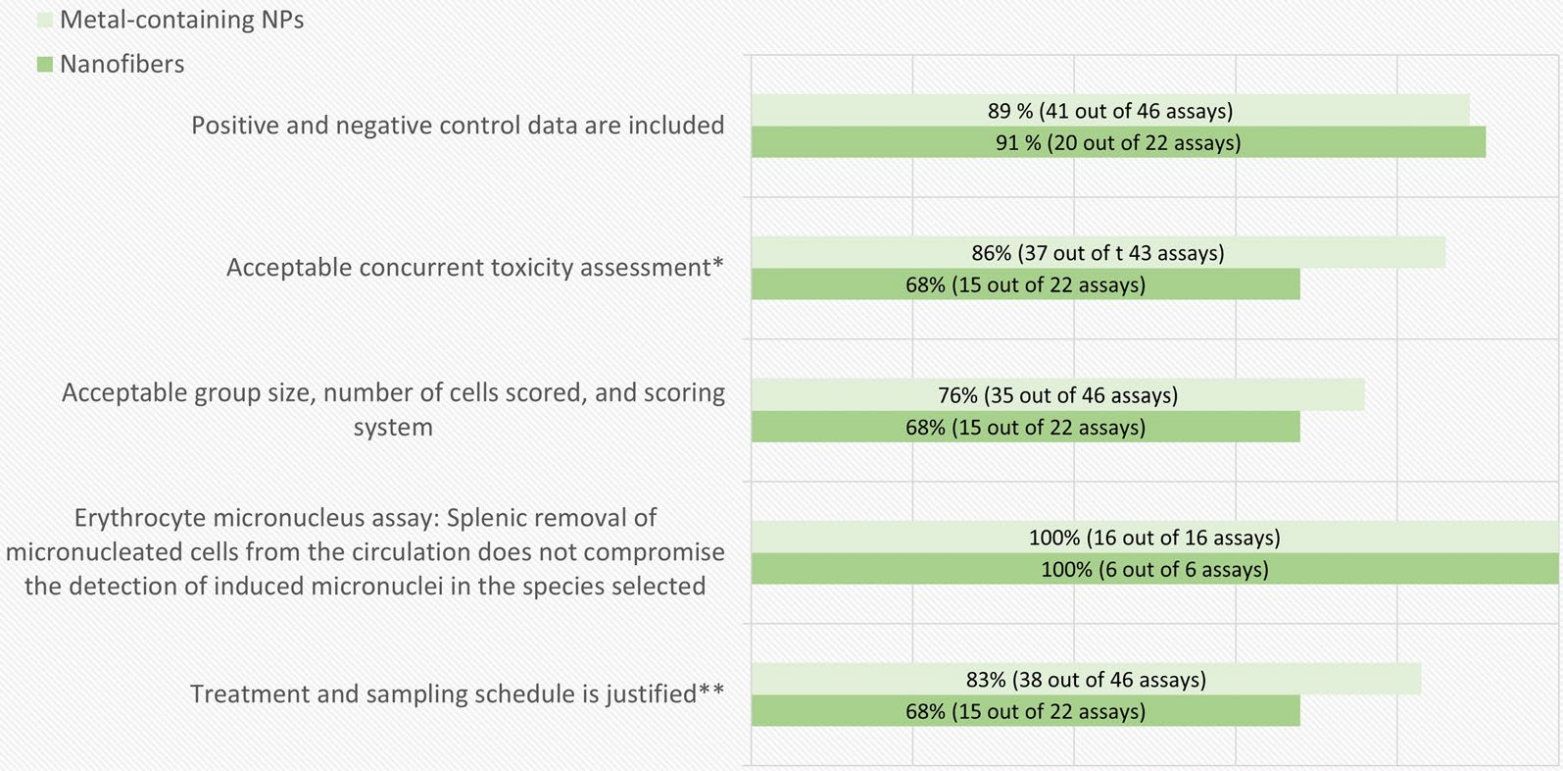
The breakdown of the in vivo genotoxicity assay-specific evaluation results by obligatory criteria. The percentage and number of assays (out of the total number of assays evaluated for each criterion) that fulfilled each assay-specific criterion is presented in each bar. *This criterion was not required for the gene mutation assay. A test that followed an older (1997) version of the corresponding OECD guideline, and had clearly positive results, was considered acceptable for micronucleus and chromosomal aberration assay. **Tests with positive results were accepted whatever treatment or sampling schedule used

A breakdown of the in vivo genotoxicity assay quality evaluation results according to the obligatory criteria is shown in Fig. 6. The most common shortcoming was a sample size that was smaller than recommended by the current OECD guidelines (sample size was revised in the 2014 version), or a draft of such guideline in case of the Pig-a assay. However, in the case of MN and CA assays, the sample size often followed the 1997 version of the OECD guidelines. In this case the test could be considered acceptable by expert judgement if the negative controls reached sufficient levels to enable reliable analysis and the test result was positive. A similar approach was applied regarding the concurrent toxicity measurement, where the only complaint was the cell number analyzed for the cytotoxicity parameter. Rare events such as chromosomal aberrations often require analysis of a larger number of metaphases (200 cells per animal) compared to what is recommended by the older (1997) version of the OECD guideline (100 cells per animal), however the data was in most cases useful even if the older version of the guideline was followed. A design with too low sample size may, however, pose a problem with interpretation of negative or weak positive results as lack of statistical power compromises the sensitivity of the test [48].

Another shortcoming concerned to the treatment and sampling schedules. A justification of a treatment schedule should optimally rely on toxicokinetic studies, which confirm the presence of the test material in the target organ at a given time point and, on the same time, take into account the transient nature of the measured phenomenon. A study using unjustified treatment schedules could be acceptable only if the test result is positive. However, in this evaluation we accepted studies that followed the OECD recommendations, although their suitability for NMs can be sometimes questionable as it may take a longer time for NMs to reach the target organ compared with soluble chemicals [5]. Especially in the case of comet assay, which is based on DNA damage that is usually repaired within hours, the bio-persistence of the nanomaterial should be confirmed, if samples are collected later than recommended.

**Fig. 7:**
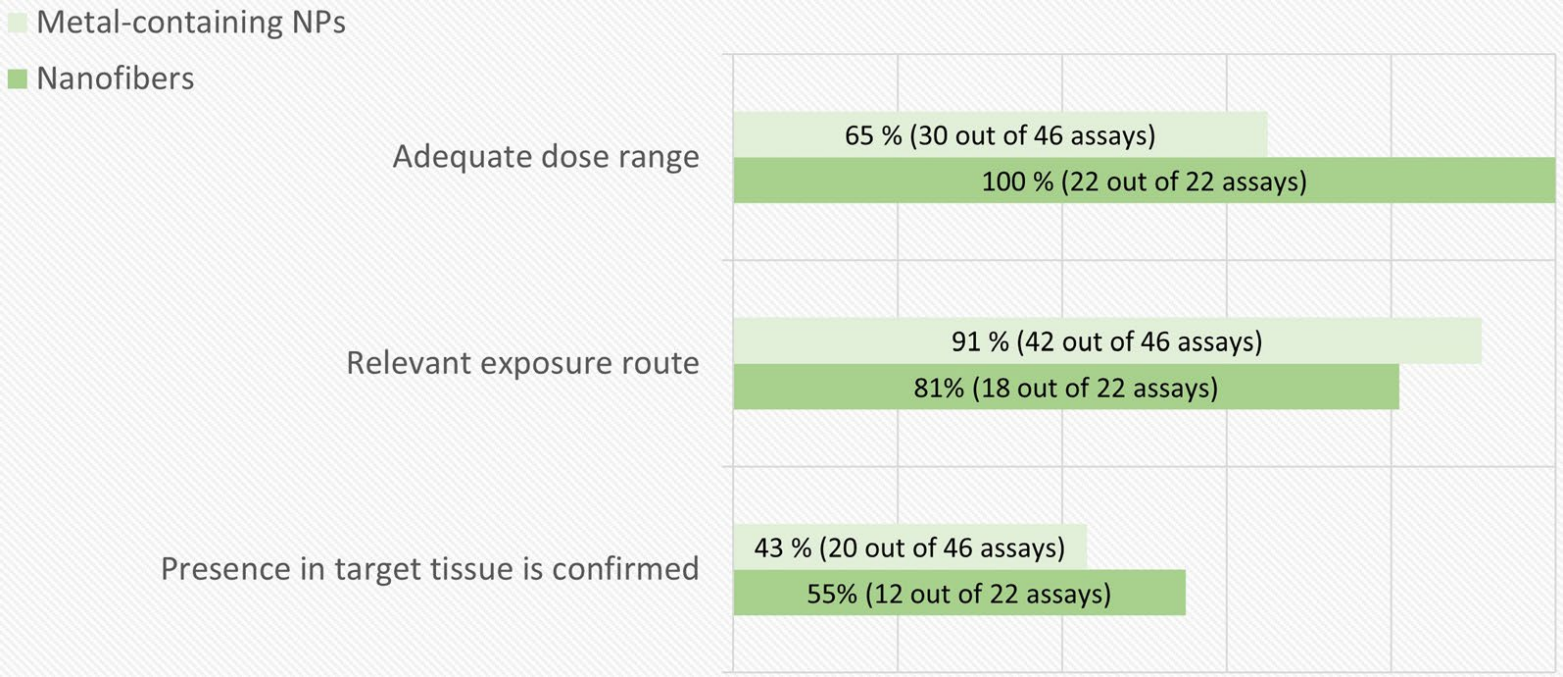
The breakdown of the in vivo genotoxicity assay-specific evaluation results by non-obligatory criteria. The percentage and number of assays (out of the total number of assays evaluated for each criterion) that fulfilled each assay-specific criterion is presented in each bar

The breakdown of the in vivo genotoxicity assay quality evaluation results according to the non-obligatory criteria is shown in Fig. 7. The main shortcoming for these criteria was the failure of confirming the presence of the material in the target tissue that, as commented in the previous paragraph, is necessary for the correct interpretation of negative outcomes. For instance, in the case of metal-containing NPs, only 3 out of 21 studies evaluated the biodistribution in all the target tissues where genotoxicity was measured, whereas 13 included some biodistribution data, but not all target tissues were measured, and 5 included no biodistribution data. Although 17 out of 21 studies included assessment of systemic genotoxicity, accumulation in bone marrow was measured in only 3 studies. In 9 out of these 17 studies, peripheral blood was used as an indicator of systemic distribution. However, as bone marrow is the target organ for the currently accepted tests for systemic genotoxicity and there may be significantly less NM available in the bone marrow compared to peripheral blood, measuring accumulation in blood may not be sufficient. In one of the studies that did consider the biodistribution in bone marrow, oral exposure to different sizes of Ag NPs led to minimal silver accumulation in the blood and especially in the bone marrow compared to other organs [49]. Unfortunately, as pointed out before, an appropriate TG for assessing the toxicokinetics of NMs is still in development [39].

Regarding the use of an adequate route of exposure, most of the studies were considered as fulfilling this criterion. Although the route of exposure should be chosen based on the realistic human exposure, it is worth noting that, according to the recommendations of the European Chemicals Agency for the safety assessment of NMs, studies are recommended to be performed via the respiratory route [36]. Only two studies, one by inhalation and the other by intratracheal instillation, explored the respiratory exposure to NMs in the case of metal-containing NPs, whereas a total of 11 studies (one by inhalation) did it in the case of nanofibers.

### Outcomes from the qualified publications

For the metal-containing NPs, 20 out of 34 in vitro publications (59%) and 15 out of 21 in vivo papers (71%) that fitted the requirements of the GUIDEnano quality assessment, also passed the assay-specific criteria put forward by the authors of the present manuscript. With respect to the nanofiber publications, the corresponding numbers were 7 out of 21 in vitro (33%) and 12 out of 18 in vivo (71%) papers. This elevated success rate observed in vivo may therefore be a result of the stricter test guidance which applies to toxicology testing in animal models as opposed to in vitro cultures. When considering the total number of papers evaluated in this study (Fig. 1), only 20 (15%) of the 137 in vitro publications and 15 (18%) of the 85 in vivo publications were considered of acceptable quality from a regulatory perspective for the metal-containing NPs. In the case of nanofibers, the corresponding numbers were 7 out of 57 evaluated in vitro publications (12%), and 12 out of 37 in vivo publications (32%).

The qualified publications covered a broad range of metal-containing NPs, including only 1–2 publications per chemical composition except for TiO_2_ and Ag NPs, for which more results were available. Conversely, the majority of the nanofiber studies concerned single- or multi-walled carbon nanotubes (SWCNT and MWCNT), and only one publication among those assessing other fibers (including nanocellulose, carbon nanofibers, imogolite, europium nanorods, cotton fibers, and graphene nanoribbons) passed the quality assessment. Most of the test results were positive, this may reflect true NM-induced genotoxicity, but may also be the result of publication bias, as negative results are usually more difficult to get published. Furthermore, more restrictive quality criteria were applied to studies reporting negative results, as shown in the previous sections (e.g., more justification for treatment schedule, cellular uptake or biodistribution is required in case of negative results).

#### TiO_2_ nanoparticles

Table S4 (Additional file 2: Table S4) summarizes the in vitro studies performed with different types of TiO_2_ NPs. The results were not consistent as different types of NPs and cell systems were used. TiO_2_ P25 AEROXIDE (also known as JRC NM-105), a 15–30 nm TiO_2_ anatase/rutile that was used as a benchmark NP in many studies, produced inconsistent results in different cell lines. P25 AEROXIDE induced a statistically significant increase in MN at 20, 50 and 100 µg/ml compared with the untreated cells, together with a significant dose–response, in bronchial epithelial BEAS-2B cells, but only when using serum-containing medium [44]. Interestingly, these authors also found that the genotoxicity results were highly dependent on the quality of dispersion. P25 AEROXIDE also induced a significant increase of MN in mouse Balb/3T3 fibroblasts, but only at the lowest tested concentration (10 µg/cm^2^) [50]. Conversely, the same material did not increase the frequency of MN in human TK6 lymphoblasts and human lymphocytes [51]. However, cellular internalization was not confirmed in the latter study. Di Bucchianico et al. [52] tested three types of TiO_2_ with different particle size and crystalline structure. Both 5–8 nm anatase (NM-100) and 22–28 nm rutile induced a significant increase of MN at 1 and 1–5 µg/ml, respectively. However, a larger 50–150 nm anatase particle (NM-103), which has a tendency of forming large aggregates, produced a negative result regardless of confirmed particle uptake. However, as recognized by the authors, cellular uptake was only assessed by flow cytometry side scatter, making impossible to distinguish whether the particles have been internalized or attached on the surface of the cells. Significant increase of MN was also induced by 50 nm TiO_2_ anatase in the epidermoid carcinoma cell line A431 [53]. Negative results were obtained with the in vitro cytokinesis-blocked NM (CBMN) assay in Caco-2 cells when testing 20–60 nm TiO_2_ anatase [54]; however, the study did not include an assessment of cellular uptake.

One study investigated the mutagenicity of < 25 nm TiO2 anatase by using the CA assay after 24, 48 and 72 h culture of human lymphocytes [55]. A significant increase of aberrations, together with a significant dose–response, was reported at 48 h culture.

TiO_2_ was the only metal-containing nanoparticle for which we found acceptable in vivo data for the respiratory route (Additional file 2: Table S5). Sprague–Dawley rats were repeatedly intratracheally instilled with P25 AEROXIDE, resulting in a significant increase of peripheral blood micronucleated erythrocytes at 35 days post-administration [56]. On the other hand, the inhalation exposure of male C57BL/6 J mice with 21 nm TiO_2_ anatase/brookite for 5 days, 4 h/d, also increased the frequency of micronuclei in peripheral blood erythrocytes [57]. Both studies also analyzed the induction of DNA damage by the comet assay. However, this assay was not considered acceptable in any of the studies due to small sample size and lacking cytotoxicity indicator. A third study with acceptable comet assay data found a significant increase of DNA damage in the liver cells of male Swiss albino mice after a 14-day repeated oral exposure to 10–100 mg/kg of 20–50 nm TiO_2_ anatase [58]. Interesting, the results were also positive when applying the enzymatic (Fpg) version of the comet assay, indicating oxidative DNA damage.

Based on the above results, certain forms of TiO_2_ NPs seem to have mutagenic potential in vivo. However, it is unclear whether these effects are only caused by secondary mechanisms of action, as the outcomes of the in vitro assays – which can only detect primary mechanisms [38]—are contradictory among particles and cellular systems. Interestingly, a recent scientific opinion of the European Food Safety Agency [59] concluded that a concern for genotoxicity of TiO_2_ particles that may be present in the food additive E 171 cannot be ruled out.

#### Ag nanoparticles

The in vitro assay quality evaluation yielded four acceptable publications on Ag, including data on pristine, citrate-coated and polyvinylpyrrolidone (PVP)-coated Ag NPs (Additional file 2: Table S6). The results appeared to depend more on the primary size than on the coating. In TK6 lymphoblasts, the MN assay results were positive for small (≤ 20 nm) Ag particles regardless of the coating, but negative or positive only at cytotoxic doses for larger 50–100 nm Ag particles [60, 61]. In L5178Y mouse lymphoma cells, on the other hand, only citrate-coated 20 nm Ag NPs gave a clear positive response, whereas with the PVP-coated and larger 50–100 nm particles, the statistically significant positive responses coincided with significant cytotoxicity [60]. In accordance with the previous, PVP-coated 42.5 nm particles also gave a negative result in bronchial epithelial BEAS-2B cells [62]. The mouse lymphoma assay results were either negative (pristine particles) or positive only at doses which also exhibit significant cytotoxicity (coated particles) [60, 63]. However, the two consistently negative studies did not confirm cellular uptake [62, 63].

In the two in vivo studies on Ag, identified as acceptable in our evaluation, different sizes of pristine, PVP-coated and silica-coated Ag particles were tested by the peripheral blood MN test (Additional file 2: Table S7). Boudreau et al*.* found no systemic genotoxicity, although the accumulation of Ag NPs in bone marrow and blood after oral gavage was smaller compared to other organs [49]. Another study by Li et al. [64] found no genotoxicity after intravenous administration of PVP- and silica-coated Ag particles. However, acceptable data was limited and only available for systemic genotoxicity. As the in vitro studies show genotoxic potential may exist in some conditions, more in vivo evidence is needed.

#### Other metal-containing nanoparticles

With the exceptions of TiO_2_ and Ag NPs, mainly 1–2 studies per chemical composition were found acceptable in the assay quality evaluation. These studies reported positive genotoxicity results with Co_3_O_4_, Cu–Zn alloy, Fe_3_O_4_, Ni, NiO, ZnO, a variety of coated quantum dots (QD), W, and WC–Co in vitro (Additional file 2: Table S8) and with CeO_2_, Cr_2_O_3_, MgO, and MnO_2_, WO_3_, and Y_2_O_3_ in vivo (Additional file 2: Table S9). Only one in vivo study with Fe_2_O_3_ reported negative results in bone marrow CA assay and peripheral blood comet assay [65]. However, only small amounts of material were found in bone marrow compared to liver, spleen, kidney, and heart, and thus testing these other organs would have been warranted.

For ZnO we found two acceptable in vitro MN tests, both of which, however, had some limitations. Senapati et al. [66] observed cellular uptake and reported positive results when treating THP-1 monocytes for 3 h, and thus, the study was considered acceptable despite the short treatment time. Zijno et al. [54] also concluded positive results in intestinal epithelial Caco-2 cells, but as the MN test only gave a positive result at highly cytotoxic doses and dose response was not tested, the result should be interpreted with care. We found no acceptable in vivo studies with ZnO NPs.

NiO NPs were tested in one study with BEAS-2B bronchial epithelial cells in vitro [47] and one oral gavage study in vivo [67]. In both studies, NiO NPs exhibited genotoxicity. In the in vivo study, NiO NPs were found in all the tested organs of female albino Wistar rats, and both local and systemic genotoxicity was systematically observed in the comet assay of peripheral blood leucocytes, liver and kidney, in the erythrocyte MN assay, and in the bone marrow CA assay [67]. As for the rest of the in vivo studies on metal-containing NPs we found acceptable in this evaluation, there was no overlap with the NMs tested in vitro.

For tungsten oxide (WO_3_) and yttrium oxide (Y_2_O_3_) NPs, two acceptable studies with positive genotoxicity results for each material were found, all conducted by the same research group. These studies included a single oral exposure with female Wistar rats, and a 28-day repeated oral exposure study with both male and female Wistar rats. Comet and MN assays for liver and peripheral blood erythrocytes, respectively, were all positive in rats treated with a single dose of Y_2_O_3_, and both single and repeated dose of WO_3_ at the highest tested dose (1000 mg/kg body weight) [68–70]. In rats treated with a repeated dose of Y_2_O_3_, all tests were positive already at 120–480 mg/kg body weight [71].

Based on the above results, many of the assessed NPs seem to be able to induce genotoxicity by primary mechanisms. In the case of ZnO, the partial overlap between genotoxic and cytotoxic doses [72], may affect the outcome of the assays. On the other hand, an occupational exposure limit (OEL) was set for nickel compounds (including NiO) as it was considered that there is a mode-of-action based threshold for these genotoxic carcinogens [73]. However, this OEL is not applicable to nano-sized particles. Although most of the NMs showing primary genotoxicity are assumed to act though indirect mechanisms (not involving direct interaction with the DNA molecule) [3, 13, 37], which can have a thresholded response [74], it is unclear whether the cut off values are similar to those of their counterpart particles.

#### Nanofibers

As concerns the in vitro publications, each of the 7 papers which qualified was investigating CNTs as the test NM (Additional file 2: Table S10). From these 7 publications, 6 focused upon the in vitro CBMN assay. Interestingly, in each occasion of the in vitro CBMN assay being utilized, the test material induced a positive response for at least one concentration. For those publications, cellular uptake was confirmed in two studies, the first of which in 2013 by Manshian and colleagues [75]. Manshian et al*.* reported significant point mutations with the *Hprt* assay as well as primary genotoxicity in MCL-5 and BEAS-2B cells respectively, following exposure to three variants of SWCNTs. These CNTs differed in length primarily, ranging from 400 to 800 nm, 1 to 3 µm and 5 to 30 µm. These CNTs were exposed to the cells over a range of concentrations up to and including 100 µg/ml.

The use of BEAS-2B cells to detect genotoxicity following CNT exposures in vitro was investigated in the work by Catalan et al. [76] and Louro and colleagues [77]. Catalan et al. [76], reported no statistically significant effects for the in vitro CBMN assay following 5, 10 and 100 µg/cm^2^ exposures. Louro and colleagues [77] conversely, showed significant data in the in vitro CBMN assay although in the human alveolar epithelial (A549) cell type. This did require fairly high concentrations of NM401 and NM402 however (with NM400 and NM403 inducing no genotoxicity), with the concentration range extending up to 150 µg/cm^2^ [77]. Tavares et al. [78], and Catalan et al*.*[55], both utilizing human blood-derived lymphocytes were both able to demonstrate significant DNA damage. Firstly, Tavares and colleagues showed that MWCNTs at just 15 µg/ml induced significant chromosomal damage using the in vitro CBMN assay, however this proved to be CNT-type dependent [78]. Catalan and colleagues reported that both SWCNTs and MWCNTs between 6 and 300 µg/ml were capable of inducing significant increase of CAs in human blood-derived lymphocytes. These effects appeared to be time dependent as the significant data findings were found following 48- and 72-h exposures [55].

For the in vivo publications which successfully passed the assay-specific criteria (Additional file 2: Table S11), only one publication focused on a material other than CNTs. Catalan et al*.* [79] investigated the effects of nano fibrillated cellulose (NFC) over a range of 10, 20, 40, 80, 200 µg/mouse, delivered via pharyngeal aspiration into female C57BI/6 mice. The authors then utilized the in vivo comet assay and the MN assay in bone marrow erythrocytes. From the investigation the authors reported dose-dependent accumulation of NFC in the bronchi and contained within macrophages. This corresponded to significant DNA damage in the comet assay, however negative findings were reported in the MN assay [79]. The remaining 11 in vivo publications which passed the assay-specific guidance all focused upon either SWCNTs or MWCNTs. From these publications, the in vivo comet assay proved to be a common technique employed to ascertain CNT genotoxicity. From the in vivo comet data, three publications reported no significant findings whereas 7 reported at least one significant dose which induced a genotoxic response. Of the three negative studies, Pothmann et al*.* [80], Christophersen et al*.* [81] and Honda et al*.* [82] all reported no significant results. All three studies utilized different rodent models, different doses of CNTs (two of which were MWCNTs), and two of the studies investigated the toxicokinetics. In the work by Pothmann and Honda and their respective teams they confirmed alveolar deposition of CNTs reaching the target tissue and still reported negative findings with the in vivo comet assay [80–82]. Where the in vivo MN assay was utilized to determine chromosome damage, five studies reported negative data whereas only two report significant dose response data. These results were published by Patlolla et al*.* in 2010 [83] and 2016 [84], who investigated the effects of MWCNTs and then SWCNTs on adult male Swiss-Webster mice via intraperitoneal exposures of 0.25, 0.5 and 0.75 mg/kg. The MN data from these studies reveal only the top two doses could induce significant chromosomal breakage, which was further supported in both cases by positive comet data and CAs [83, 84].

In conclusion from the qualified in vitro papers, we can deduce that nanofiber genotoxicity can be shown in several key cell lines at low and high concentrations, following both acute (24 h or less) and slightly longer exposures of 48- and 72-h. Furthermore, in the studies which utilized uptake as part of the methodology, cellular internalization had been confirmed using TEM. These findings however do not seem to discriminate between SWCNTs and MWCNTs in terms of mode of action inducing their genotoxic effects. It appears more likely that their geometry and fiber paradigm are the primary factors driving their genotoxicity, which has been supported both in the literature and the qualified publications of this study. Where the qualified in vivo publications were concerned the majority of the data indicated negative responses even at when high, but still adequate doses were used. Additionally, when the in vivo comet assay was performed, 11 of the studies reported the test material was present in the target tissue thus demonstrating that the toxicity (or lack of toxicity) was reliably reported. From the qualified studies, the data is not conclusive enough to strictly classify nanofibers (the majority of which are CNTs) as genotoxic.

## Database search

The NanoinformaTIX database is an instance of the eNanoMapper database (https://search.data.enanomapper.net/projects/nanoinformatix/), which collects data from 8 EU initiatives plus the US caNanoLab. This database also collects data from the NanoinformaTIX project, currently still on going and is periodically updated. The information in the database is therefore dynamic and should be checked regularly. Hence, information provided in this review represents a snapshot of a particular time.

Regarding the present study, the NanoInformaTIX instance was searched for genotoxicity entries selecting relevant filters. All entries regarding genotoxicity were considered- whether they used validated or non-validated assays- as the main idea was to provide an overview of how genotoxicity was covered by the database, as well as the amount of data a future stakeholder could benefit from. Filtering was performed selecting under the nanomaterial menu “metal” OR “carbon-based” materials. The latter being expected to capture similar materials as the ones obtained in the literature review when searching by “nanofibers”, as most of them were CNTs. Results were subjected to a second filtering step under the protocols tab. The following were selected: "comet OR comet (ox. DNA damage) OR comet (primary DNA damage) OR comet (Fpg) OR comet (NET-Fpg) OR comet-SB, OR genotoxicity OR cell transformation assay (CTA) OR DNA strand breaks OR in vitro micronucleus OR in vivo comet assay OR micronucleus OR mouse lymphoma L5178Y/TK ± assay OR genetic toxicity in vivo* OR* HCA OR pH2Ax4h OR pH2Ax4h in vivo". To the best of our knowledge, this broad range of entries covers all possible DNA damage-related data that could be available in the database.

### Genotoxicity data available in the NanoInformaTIX instance

After searching in the NanoInformaTIX instance, the identified relevant NMs for which genotoxicity entries were found were CNTs, TiO_2_, ZnO, BaSO_4_, Ag, Fe_3_O_4_ and ZrO_2_. As shown in Additional file 2: Fig. S1, the rate of data entries regarding genotoxicity compared to the overall toxicity data entries per material ranged from 7% (Ag) to 40% (ZrO_2_). Therefore, the NanoInformaTIX instance contains a substantial amount of genotoxicity information. A total of 41 data entries related to genotoxicity were identified in the NanoInformaTIX instance. As shown in Additional file 2: Fig. S2, the distribution of the genotoxicity-related data entries per material was as follows: CNT (34%), TiO_2_ (29%), ZnO (12%), Ag (10%), BaSO4 (2%), F_3_O_4_ (2%), ZrO_2_ (2%). Furthermore, different nanoforms were identified under the general nanomaterial names and those are shown in the Additional file 2: Table S12. As expected, these numbers correlate with the predominance of the NMs found in the literature search.

Regarding assays, various names were provided per assay (as shown in Additional file 2: Table S13). This is because it is up to the laboratory that enters the data to decide how to name each assay. In some cases, the existence of different names is justified as they refer to different variations of the same assay. For instance, the formamidopyrimidine DNA glycosylase (Fpg)-modified comet assay is a modification of the standard comet assay where the addition of repair enzymes allows measuring oxidatively damaged DNA [85]. In other cases, however, different names call for the same approach (e.g., in vitro micronucleus and micronucleus). This situation in some instances hampers the result overview, since the exact name must be entered in the searching engine, and thus, several possibilities were included in this review to get a full picture of the information collected in the database.

**Fig. 8:**
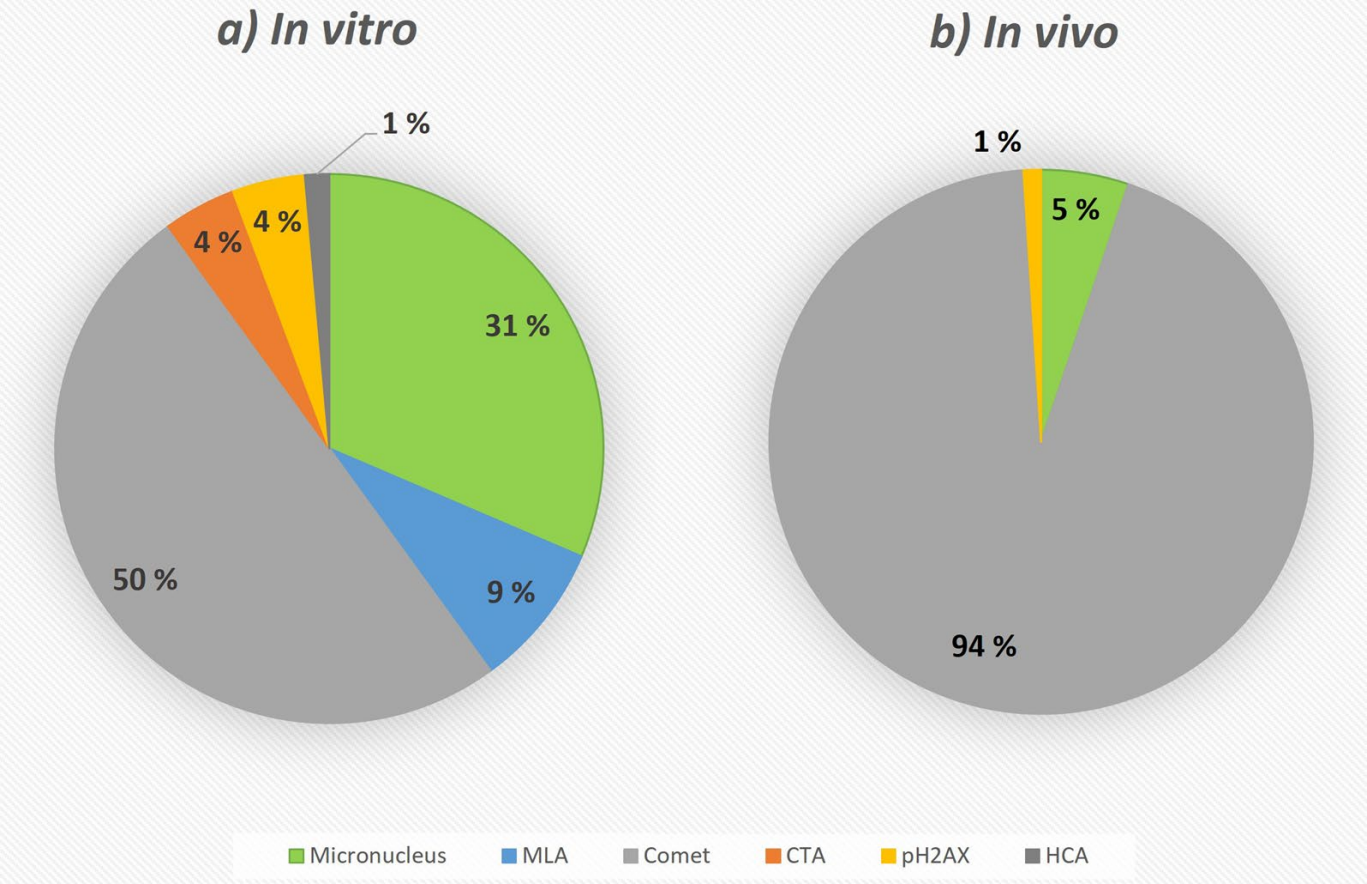
Overview of the percentage of the in vitro (**a**) and in vivo (**b**) genotoxicity assays entries found in the NanoInformaTIX instance (MLA: mouse lymphoma assay; CTA: cell transformation assay; pH2AX: phosphorylation of H2AX assay; HCA: high content analyses)

Regarding the in vitro studies identified in the NanoInformaTIX database (Fig. 8a), at least half of the assays identified represented different versions of the comet assay (50%), followed by the micronucleus (31%) and the mouse lymphoma (9%) assays. Although the in vitro comet assay was not considered in the literature search because it is not a validated assay [13], it has been reported to be the most used assay among peer-reviewed publications [3]. The second most usually reported is the MN assay, in agreement with the databases. However, no data on CA were found in the database, despite existing publications using this assay. Phosphorylation of the Ser-139 residue of the histone variant H2AX (4%) as well as of other protein residues (1%) can also be used as a biomarker for genotoxicity [86]. Those studies were also identified in the database but to a lesser extent and were mainly performed by one particular institution [87]. These specific studies were part of an array of studies which included cell count, evaluation of nuclear size, nuclear intensity, active Caspase-3, gH2AX, phospho-p53, phospho-ATM, and cell count through Ki-67, and hence were covered also under the High Content Analysis entry (HCA). On the other hand, the cell transformation assays (CTA, 4%) are not assessing genotoxicity, but carcinogenicity. However, some of the initiating events detected by this assay correspond to mutagenic events [88]. Likewise, the most popular in vivo method was the comet assay, which corresponded to 94% of all in vivo studies (Fig. 8b). In fact, the so-called “DNA strand breaks” assay, although reported separately, also refers to the comet assay. On the other hand, the rest of the in vivo entries corresponded to the micronucleus and the phosphorylation of H2AX assays (5 and 1%, respectively). As with the in vitro assays, no data on CA were found, despite studies using this assay were reported in the literature search.

### Quality of the data available in the NanoInformaTIX instance

One main issue which may prevent stakeholders from using information from databases is generally the potential uncertainty about the quality of stored data. Hence, and in line with the work described in previous sections of this review, the NanoInformaTIX instance was also reviewed following the questions addressed to fulfil the S score. Most of the required information could be easily found and is reviewed in Additional file 2: Table S14, where the questions addressed and a summary of the outcomes for each question are described.

As a summary and regarding the S score, it can be concluded that NMs were generally well characterized by different PC methodologies. For instance, particle size was provided based on different methods, such as TEM, Small Angle X-ray Scattering (SAXS), Wide Angle X-ray Scattering (WAXS). The only shortcomings were generally a lack of endotoxin contamination data and a missing link between PC characterization and toxicological results. The findings contrast with the lack of complete characterization reported for peer-reviewed publications. One reason explaining this discrepancy may be that most of the data included in the databases have their origin in strong consortia, which have joined efforts to provide a robust characterization. Nevertheless to an external user it was not clear how to link PC results to a particular toxicological experiment (and the same applies to genotoxicity assessment).

Likewise, the information retrieved was reviewed to evaluate if it fulfilled the K score in vitro (Additional file 2: Table S15) and in vivo (Additional file 2: Table S16). In general, the existing data fitted most of the K score criteria. However, as happened with the PC characterization, (cyto)toxicity data are neither linked to the genotoxicity data produced in the same experiments, which hampers the correct interpretation of the genotoxicity outcomes. As there was no selection towards validated methods, no assay-specific criteria were applied in this case.

### Challenges retrieving data from the NanoInformaTIX instance

One main issue while approaching the databases is that most of them were not open to external stakeholders, this may be already an issue preventing their wide acceptability and use. Secondly, even if the searching strings were clear, several names were found to indicate one given assay and, since searches are not flexible, one needs to select different possibilities to cover all potential entries for a given assay. This issue has been identified by the different projects contributing data to the eNanoMapper database and is currently being addressed in different European initiatives [89]. Zooming into the retrieved assays, data provided were well structured with most entries meeting the requirements of the S and K score, yet unfortunately S and K score-related data belonging to the same experiments were not linked, or at least this was not obvious to the user, therefore it was impossible to assess the PC status of a particular material prior, during and after the experiment, as recommended by e.g., the NANoREG project. This information was previously imposed to facilitate interlaboratory comparisons and efforts should be made in this direction to maximize the use of the database entries. Likewise, experiments were not linked regarding cytotoxicity and genotoxicity outcomes, of if they were, it was not obvious to the reader. As a result, currently it is not possible to identify subtoxic concentrations which will assist in genotoxicity experimental planning.

Although an embargo period is usually set before making the data openly available, to allow researchers to publish their results, no link to those publications is usually provided in the databases. Hence, it is not possible to track which information available at the database do exist or not in the published literature. Such interconnection could help the studies to be qualified and used, as some of the missing information (e.g., details on PC properties not properly reported in the publications) may be available in the database. In fact, fulfilling the S score criteria, which was a key shortcoming among published literature, does not seem to be an issue for databases.

Another issue that may preclude the use of databases by regulators is the fact that the information contained in them consists of raw data. Therefore, the use of this type of information may somehow be restricted to experts on the field, as no outcomes of the genotoxicity assessment are provided. Furthermore, there is neither possible to know the ratio of positive *vs.* negative results present, making the comparison with the published information impossible.

## Recommendations for designing genotoxicity studies of nanomaterials with regulatory relevance

According to our quality assessment, only 17% of the published information on the genotoxicity of NMs (54 papers out of a total 316 evaluated publications) would be considered as reliable and relevant by a regulator, given these publications fulfill the quality criteria. This percentage is especially small having in mind that our search was restricted to studies using validated methods and that the S score applied is only assessing whether some PC characteristics have been analyzed, without evaluating the quality of such characterization. These findings call for a reflection on how genotoxicity studies should be designed and performed to provide data that are not only scientifically relevant, but also fit to the regulatory requirements. A set of recommendations is summarized in Table 4.

**Table 4:** Recommendations for designing genotoxicity studies of nanomaterials with regulatory relevance

**•** A full set of physico-chemical characteristics should be reported in the same publication with the genotoxicity results
**•** The test system characteristics should be well understood, described, and selection justified
**•** Concurrent cytotoxicity measurements should be conducted using the parameters recommended by the latest version of the OECD test guidelines (TGs)
**•** In addition to the current OECD TGs, nano-specific requirements already adopted by the regulatory agencies should be checked and followed; e.g., the use of a delayed Cytochalasin-B treatment or the need of one cell cycle length treatment with nanomaterials with the in vitro micronucleus assay
**•** Following the OECD guideline with the inclusion of additional wash steps is recommended for the *Hprt* gene mutation test
**•** A justification of an in vivo treatment schedule should rely on evidence which confirms the presence of the test material in the target organ at a given time point and take into account the transient nature of the measured phenomenon
**•** Harmonized names of the assays and ways of reporting results should be used when entering genotoxicity data into the databases
**•** Non yet validated assays, performed with complex and realistic experimental models, that can provide information on the genotoxicity mechanisms of action of nanomaterials are urgently needed

Most of the rejected studies failed in providing a complete PC characterization of the tested materials. The completeness of PC characterization in the published literature has increased in the recent years in parallel with the evidence of the key role that those properties may play on the toxicological response of the NMs [90], and how grouping strategies could benefit of knowing such relationships [91]. However, no standardized methods have been agreed for measuring some of the properties [39]. On the other hand, the expertise and equipment required for some of them are out of the capacities of most toxicology laboratories. Consequently, many well-performed genotoxicity studies were rejected during our exercise due to the limited characterization information they could provide. Factors which will help future research to elucidate NM genotoxicity would be reporting on the full PC features of the materials in the publication. Common characterization efforts among different partners, as performed in several ongoing projects and reflected in the evaluated databases, can contribute to overcome the lack of characterization resources at the individual lab level.

Lack of reliability and relevance, evaluated through the K score and the assay-specific criteria, concerned 16–27% of the articles with appropriate characterization. There may be several reasons explaining this phenomenon. First, it is worth mentioning that the focus of some of the rejected articles was not the assessment of genotoxicity. For instance, comparisons between nano and non-nano sized particles are sometimes done using only one or two doses. Although scientifically sound, the lack of a third dose led to the exclusion of these types of studies. A typical shortcoming in all the genotoxicity assays was either the lack of concurrent cytotoxicity data or use of a parameter, which is not among the parameters recommended by the OECD. In some cases, cytotoxicity data from a separate experiment was shown, but this is inadvisable unless there is clear evidence that the experiments have a good repeatability and there are no other differences between the experimental conditions of the assays. Therefore, it is strongly advised that before starting the experiments, researchers check the guidelines and determine what additional parameters should be included to fulfill their research goals and, at the same time, provide an adequate assessment of the genotoxic effects.

Secondly, although most of the existing guidelines are applicable to NMs, some of them still require some adaptations. Some of the compulsory assay-specific criteria, e.g., delayed Cyt-B treatment or the treatment for at least one cell cycle, are not set for NMs within the current OECD guidelines. Therefore, some studies have been rejected despite fitting to the guidelines’ requirements. Although some of the nano-specific requirements have already been adopted by the regulatory agencies [36, 40], the researcher should know where to find the appropriate information. There is an urgent need for adapting and updating OECD TGs for assessing the genotoxicity of NMs. Several efforts are currently approaching this issue under the umbrella of the OECD programme on NMs [39].

It is worth mentioning the lack of investigations performed with mammalian gene mutation assays. Only 8 in vitro and 3 in vivo tests were done using these methods. From those, only half of the in vitro and none of the in vivo assays passed the quality assessment. Current efforts within the EU H2020 RiskGONE project have worked towards harmonization of the *Hprt* gene mutation assay protocol to suit the evaluation of NMs. Essentially, the OECD TG for this method is appropriate, however, a minor adaptation to include additional wash steps to better remove the test article following the exposure period is recommended.

Besides validated genotoxicity TGs, other guidelines related to NMs’ exposure are also needed to allow correct interpretation of the genotoxicity outcomes. This is the case for the toxicokinetics guideline for NMs, which is currently in progress [39]. Although toxicokinetics evaluation is not required as part of the regulatory information requirements, future in vivo genotoxicity studies with NMs should confirm the presence of the material in the target tissue, especially if the outcomes are negative. In that sense, some of the current in vivo genotoxicity guidelines (e.g., OECD TG 474 on the mammalian erythrocyte micronucleus assay) may also need to be adapted as one main concern with NMs is whether they can reach the target tissues when using the treatment schedules that have been optimized for soluble chemicals [5]. In parallel, validation of new assays able to assess the effects of the NMs at the site of contact (e.g., micronuclei in pulmonary cells) should be achieved to ensure an appropriate mutagenicity assessment of the NMs.

Regarding databases, there is a need for harmonizing assays’ names and the way of reporting results. It is a challenging effort to harmonize a large number of entries collected from different laboratories in different projects such as NanoInformaTIX, and, at present, for an external user it is a challenging task to make sure all entries of interest are retrieved. Nonetheless, the authors are fully aware that harmonization efforts are currently on-going under the eNanoMapper database (Jeliazkova, *personal communication*). The database is also very dynamic and data uploads take place on a routine basis. Data retrieved from the eNanoMapper database generally contained all information required by the S and K scores indicating that the database may be used as a source of good quality data for academia, industry, or regulators alike. A minor drawback may be represented by the masks or interface used, which sometimes may not be optimal for the external user to find relevant information. On the other hand, the data outputs collected from the database may not be suitable to risk assessors, who may not necessarily be familiar with the tests and their resulting values but need to interpret the results. On this front, the database may benefit from an extra column entitled “results interpretation”. In the future, databases are also recommended to include a link to the published publication(s) where these data have been generated or re-used. In the case of negative outcomes, with less possibilities of getting published, new tools should be developed to allow stakeholders (including regulators) to analyze the data without having a high genotoxicity expertise.

As stated within several points throughout this review, we have focused on the validated assays that are used to fulfill the regulatory information requirements. However, regulators are also in the need of mechanistic studies that could contribute to investigating the genotoxic mechanisms of action of the NMs. The current paradigm on genotoxic carcinogens considers that only DNA-reactive substances have a non-thresholded mode of action [92, 93]. Co-culture systems involving inflammatory and target cells can contribute to discriminate between primary and secondary genotoxic mechanisms without the need of animal studies and have been already used with NMs [38, 94, 95]. The use of centromeric probes in combination with the MN assay can allow identified aneugenic materials, which are also assumed to have a threshold response [96].

Although not addressed in the quality evaluation approach presented in this review, for the purpose of increasing confidence in the genotoxicity studies it is also useful to understand the test system in detail, present sound justification for selecting it, know how it responds to a positive particle, chemical, and assay-specific controls, and monitor its characteristics to detect possible anomalies that could affect the test outcome [97]. At the moment it is not possible to evaluate all aspects of the study design as there is no clear consensus on the approaches that should be considered the most relevant. It can be considered important to be able to show the genotoxic potential of a NM regardless of how representative the test system or study design is, but on the other hand, more complex, representative, and realistic models are needed to increase the predictiveness and reduce the uncertainty, particularly related to non-animal models [98]. However, for regulatory purposes, the methods need to be reproducible and validated. International efforts to develop such methods are ongoing, but shortage of in vivo data and resources needed for validation of the methods create challenges for rapid implementation. In future, adverse outcome pathways may provide a faster and more cost-efficient strategy to increase confidence in non-animal methods.

## Conclusions

Most published studies on the mutagenic effects of NMs do not fit to the requirements to be considered as complete, reliable, and relevant from a regulatory perspective. The most important barrier between the regulators and the researchers is the lack of nano-specific guidelines for both genotoxicity assays and supporting information, such as the PC characterization and toxicokinetics of NMs. In addition, validated new methods that could overcome some of the limitations of the current guidelines, and provide more mechanistic information are urgently needed. Furthermore, databases may contain relevant information, especially regarding negative outcomes, that could support regulatory assessment. However, the lack of harmonization in the nomenclature used together with the difficulties to find and link the data, currently preclude their use and implementation by regulators.

## Supplementary Information

**Additional file 1.** Reproduction of the scores and criteria used in the GUIDEnano quality approach.

**Additional file 2.** Outcomes of the qualified publication and evaluation of the NanoInformaTIX database instance.

## Data Availability

All data generated or analyzed during this study are included in this published article [and its Additional files 1 and 2].
