# Correction: a systematic quality evaluation and review of nanomaterial genotoxicity studies: a regulatory perspective

**DOI:** 10.1186/s12989-022-00509-3

**Published:** 2022-12-28

**Authors:** Kirsi M. Siivola, Michael J. Burgum, Blanca Suárez-Merino, Martin J. D. Clift, Shareen H. Doak, Julia Catalán

**Affiliations:** 1 Finnish Institute of Occupational Health, Box 40, 00032 Työterveyslaitos, Helsinki, Finland; 2 In Vitro Toxicology Group, Faculty of Medicine, Health and Life Sciences, Institute of Life Sciences, Swansea University Medical School, SA2 8PP Singleton Park, Swansea, Wales, UK; 3 TEMAS Solutions GmbH, 5212 Hausen, Switzerland; 4 Department of Anatomy Embryology and Genetics, University of Zaragoza, 50013 Zaragoza, Spain

**Correction to: Particle and Fibre Toxicology (2022) 19:59**. 10.1186/s12989-022-00499-2

Following publication of the original article [1], the authors reported an error in the name of the first author, it should change from “Kirsi K. Siivola” to “Kirsi M. Siivola”.

The correct author name has been provided in this Correction.

The original article [1] has been corrected.
