# Supplementary material for: A systematic quality evaluation and review of nanomaterial genotoxicity studies: a regulatory perspective
Source: Part Fibre Toxicol. 2022 Sep 14;19:59. doi: 10.1186/s12989-022-00499-2 (PMC9472411; doi:10.1186/s12989-022-00499-2)
Supplement: Supplementary file 1 — Additional file 1. Reproduction of the scores and criteria used in the GUIDEnano quality approach. [file 12989_2022_499_MOESM1_ESM.pdf]

Additional file 1

A systematic quality evaluation and review of nanomaterial genotoxicity studies – a regulatory perspective

Siivola KM[1], Burgum MJ[2], Suárez-Merino B[3], Clift MJD[2], Doak SH[2] and Catalán J[1,4]

[1]Finnish Institute of Occupational Health, Box 40, Työterveyslaitos, 00032 Helsinki, Finland

[2]In Vitro Toxicology Group, Faculty of Medicine, Health and Life Sciences, Institute of Life Sciences, Swansea University Medical School, Singleton Park, Swansea, SA2 8PP, Wales, UK

[3]TEMAS Solutions GmbH, 5212 Hausen, Switzerland

[4]Department of Anatomy Embryology and Genetics, University of Zaragoza, 50.013 Zaragoza, Spain

Table S1. Substance (S) score for chemical substances and nanomaterials in the GUIDEnano quality approach (compulsory criteria are identified as red questions). Reprinted from Ref. [16]. Copyright 2018 Royal Society of Chemistry.

		Score	Comments
	**Substance (chemical or nanomaterial) characterization:**		**1)** In peer-reviewed publications, for some methodological aspects, authors may refer to previous publications. This should not be considered as missing information. The cited publication should be considered. **2)** When the assays are developed according to a guideline (USEPA, OECD, ASTM), without modifications, even if the information in relation with the chemical characterization is missing, we can assume that the specific question has been addressed. **3)** When the source of substance is well identified, the evaluator should try to find data related to the characterization given by the supplier in its pristine form for the nanomaterial.
1	Was the test substance identified?	0 or 1	Chemical name, CAS number and/or chemical composition/structure/surface chemistry including coating
2	Is information on the source/origin of the substance given?	0 or 1	
3	Is purity (concentration) of the substance given?	0 or 1	Purity refers to the concentration of the chemical or nanomaterial in the powder or in the solution/dispersion
4	Is endotoxin content of the substance given?	0 or 1	This question is not applicable for ecotoxicity studies
5	Were impurities stated?	0 or 1	
6	Was the substance concentration measured in the exposure medium?	0 or 1	
7	When the substance is a nanoparticle (NP) were protocols of dispersion and characterization in the exposure medium identified? Or, were protocols of preparation of exposure medium stated?	0 or 1	Protocols established by NANoREG or any other Project or agency. If the substance is not a NP or a dispersion step is not needed (i.e. NP aerosols) give a score of 1.
8	Was the stability of the substance concentration measured during the exposure period?	0 or 1	Stability of test compound may be assumed in *in vivo* human toxicity studies where solutions of the test item were prepared freshly prior to each administration during the exposure period.
9	Are doses administered or concentrations in exposure media given?	0 or 1	The tested doses or concentrations should be indicated or at least the range and number of concentrations with the dilution factor
10	Was the type of test medium or vehicle used stated?	0 or 1	If the compartment is sewage treatment plant give a score of 1.
11	For ecotoxicity studies, were mandatory exposure medium conditions measured?	0 or 1	Mandatory parameters depend on the compartment: 1) Freshwater and marine: pH, temperature and dissolved oxygen (fish and daphnia), pH (algae); 2) Soil: pH and organic matter; 3) Sediment: sediment TOC and pH of the overlying water; 4) Not applicable for sewage treatment plant compartment. When medium preparation is reported following guidelines protocols (i.e. OECD, etc.), unmodified, the evaluator should give a score of 1 to this point.
12	For ecotoxicity studies, were any other exposure medium conditions measured?	0 or 1	Optional parameters depend on the compartment: 1) Freshwater and marine: alkalinity, organic carbon concentration and/or hardness (and salinity/conductivity for marine); 2) Soil: cation exchange capacity, water holding capacity, textural classification and/or any other relevant parameter; 3) Sediment: Salinity/conductivity of liquid phase, textural classification, cation exchange capacity, saturation volume, dissolved oxygen concentration, physicochemical characterization of the overlying water and/or any other relevant parameters; 4) Not applicable for sewage treatment plant compartment. When medium preparation is reported following the guidelines protocols (i.e. OECD, etc.), unmodified, the evaluator should give a score of 1 to this point.

Table S1. Substance (S) score for chemical substances and nanomaterials in the GUIDEnano quality approach (compulsory criteria are identified as red questions). Reprinted from Ref. [16]. Copyright 2018 Royal Society of Chemistry.

		Score	Comments
	**Other specific nanomaterial characterization:**		If the substance is not a nanoparticle give 1 to all of the parameters
	**A: Pristine nanoparticle**		
13	Size	0 or 1	
14	Surface area	0 or 1	
15	Surface charge	0 or 1	
16	Shape	0 or 1	
17	Other relevant information (i.e. crystal structure, solubility, magnetic properties, acidity/basicity, redox potential, catalysis, photosensitivity, hydrophobicity, radical production capacity, Kow, etc.)	0 or 1	
	**B: Nanoparticle in the exposure medium**		
18	Size at the start or at the end of the exposure period	0 or 1	The size of the single nanoparticle, if it is the case in the exposure medium or as aggregate/agglomerate should be provided.
19	Surface charge	0 or 1	
20	Other relevant information (i.e. ion release, solubility, shape, etc.)	0 or 1	In case of aerosols, give a score of 1.
	**S score**		
	**Total**	17	**For sewage treatment plant compartment.**
	less than 11 questions answered YES or no all red questions answered YES	S3	characterization of the substance not acceptable
	11 to 14 questions answered YES and all red questions answered YES	S2	characterization of the substance acceptable
	15-17 questions answered YES and all red questions answered YES	S1	very good acceptability of the characterization of the substance
	**Total**	18	**For human *in vitro* and *in vivo* toxicity studies**
	less than 11 questions answered YES or no all red questions answered YES	S3	characterization of the substance not acceptable
	11 to 15 questions answered YES and all red questions answered YES	S2	characterization of the substance acceptable
	16-18 questions answered YES and all red questions answered YES	S1	very good acceptability of the characterization of the substance
	**Total**	19	**For aquatic, soil and sediment compartments**
	less than 12 questions answered YES or no all red questions answered YES	S3	characterization of the substance not acceptable
	12 to 16 questions answered YES and all red questions answered YES	S2	characterization of the substance acceptable
	17-19 questions answered YES and all red questions answered YES	S1	very good acceptability of the characterization of the substance

Table S2. Questions for *in vitro* toxicity studies and reliability of the study (K score for *in vitro* toxicity studies) in the GUIDEnano quality approach (compulsory criteria are identified as red questions). Reprinted from Ref. [16]. Copyright 2018 Royal Society of Chemistry.

		Score	Comment
	**Organism characterization**		In peer-reviewed publications for some methodological aspects, authors may refer to previous publications. This should not be considered as missing information. The cited publication should be considered.
1	Is the cell model or organism given?	0 or 1	Type of cells: primary cells, cell lines, reconstructed tissue, isolated (parts of) organs, bacteria, yeast cells.
2	Is information given on the source/origin of the test system?	0 or 1	Examples for such information are: lab/scientist providing cell lines; commercial provider of test systems; origin of primary cells, organism, etc.
	**Study design description**		
3	Are necessary information on test system properties, and on conditions of cultivation and maintenance given?	0 or 1	Necessary information on the test system and on cultivation conditions may be e.g.: contamination control; type/composition of media; use of serum/antibiotics, etc; for commercial test systems: viability, response to reference substances or other quality information provided by the supplier of the system.
4	Is the method of administration given (see explanations for details)?	0 or 1	Additional information may be necessary for complete description of the method: e.g. cell density used, sample volume, well surface, vehicle or solvent used; maximum concentration of solvent; explanation of specific conditions applying during exposure (static, dynamic, with light, darkness…); application of new medium on or after exposure.
5	Are duration of exposure as well as time-points of observations explained?	0 or 1	Time points of observations may not be mentioned when the experimental set-up makes clear that observation takes place immediately after end of exposure (considered sufficient). Please check also figures and tables for respective information.
6	Were negative and positive controls included (where and when needed)?	0 or 1	Negative controls may consist of untreated test system or test system treated with solvent/vehicle.
7	Is the number of replicates (or complete repetitions of experiment) given?	0 or 1	
	**Study results documentation**		
8	Are the study endpoint(s) and their method(s) of determination clearly described?	0 or 1	
9	Have the results been analyzed using statistical methods?	0 or 1	Statistical methods should be suitable for the dataset under analysis and the goal of the analysis. In case statistical methods are provided but the evaluator is not able to judge the suitability of the statistical methods, this question should be scored by 1.
	**K score**		
	**Total**	9	
	not all red questions answered YES	K3	study not reliable
	7 questions answered YES and all red questions answered YES	K2	study reliable with restrictions
	8-9 questions answered YES and all red questions answered YES	K1	study reliable without restrictions

Table S3. Questions for *in vivo* toxicity studies and reliability of the study (K score for *in vivo* toxicity studies) in the GUIDEnano quality approach (compulsory criteria are identified as red questions). Reprinted from Ref. [16]. Copyright 2018 Royal Society of Chemistry.

		Score	Comment
	**Organism characterization**		In peer-reviewed publications for some methodological aspects, authors may refer to previous publications. This should not be considered as missing information. The cited publication should be considered.
1	Is the species given?	0 or 1	
2	Is the sex of the test organism given?	0 or 1	
3	Is information given on the strain of test animals?	0 or 1	Other specifications, which may be important to know in the case of specific study types, are, for example,<br>- SPF (specific pathogen free) status of animals<br>- or the type of genetic modifications in knock-out/in animals
4	Is age or body weight of the test organisms at the start of the study given?	0 or 1	
5	Is information given on the housing or feeding conditions?	0 or 1	Information may consist of:<br>- temperature,<br>- humidity,<br>- light-dark cycles,<br>- information on diet and<br>- number of animals per cage.
	**Study design description**		
6	Is the administration route given?	0 or 1	
7	Are frequency and duration of exposure as well as time-points of observations explained?	0 or 1	Time-points of observations may not be mentioned when the experimental set-up makes clear that observation takes place immediately after end of exposure (considered sufficient). Please check also figures and tables for respective information.
8	Were negative (where required) and positive controls (where required) included (give point also, when absent but not required, see explanations for study types and their respective requirements on controls)?	0 or 1	For most in vivo studies, positive controls are not required. They are required in guidelines for the following study types:<br>- delayed neurotoxicity following acute exposure,<br>- local lymph node assay (skin sensitization),<br>- uterotrophic bioassay in rats,<br>- tests on genotoxicity in vivo: erythrocyte micronucleus test, bone marrow chromosomal aberration test, rodent dominant lethal test, spermatogonial chromosome aberration test , mouse spot test, mouse heritable translocation assay, unscheduled DNA synthesis test in vivo.<br>Negative controls are required for most in vivo studies and may consist of untreated animals or solvent, vehicle or sham-treated animals.
9	Is the number of animals per group given?	0 or 1	
10	Are sufficient details of the administration scheme given to judge the study (see explanation for examples)?	0 or 1	Necessary administration details may consist of information<br>- on dilution of the test item in diet, in vehicle, solvent,<br>- on the total volume applied by gavage,<br>- homogeneity of application media (e.g. feed),<br>- preparation of aerosol or atmospheres in inhalation studies,<br>- handling of animals under treatment (e.g. in nose-only studies),<br>- type of occlusion and exposed skin area in dermal exposure studies,<br>- measures taken against volatilization, etc.
	**Study results documentation**		
11	Have the study methods been described?	0 or 1	
12	Have the results been analyzed using statistical methods?	0 or 1	Statistical methods should be suitable for the dataset under analysis and the goal of the analysis. In case statistical methods are provided but the evaluator is not able to judge the suitability of the statistical methods, this question should be scored by 1.
	**K score** **Total**	12	
	less than 7 questions answered YES or not all red questions answered YES	K3	study not reliable
	7 to 10 questions answered YES and all red questions answered YES	K2	study reliable with restrictions
	11-12 questions answered YES and all red questions answered YES	K1	study reliable without restrictions

References

(as numbered in the main manuscript)

16. Fernández-Cruz ML, Hernández-Moreno D, Catalán J, Cross RK, Stockmann-Juvala H, Cabellos J, et al. Quality evaluation of human and environmental toxicity studies performed with nanomaterials – the GUIDEnano approach. Environmental Science: Nano. 2018;5(2):381-97, doi:10.1039/C7EN00716G.
