# Supplementary material for: A systematic quality evaluation and review of nanomaterial genotoxicity studies: a regulatory perspective
Source: Part Fibre Toxicol. 2022 Sep 14;19:59. doi: 10.1186/s12989-022-00499-2 (PMC9472411; doi:10.1186/s12989-022-00499-2)
Supplement: Supplementary file 2 — Additional file 2. Outcomes of the qualified publication and evaluation of the NanoInformaTIX database instance. [file 12989_2022_499_MOESM2_ESM.pdf]

*Additional file 2*

# A systematic quality evaluation and review of nanomaterial genotoxicity studies – a regulatory perspective

Siivola KM[1], Burgum MJ[2], Suárez-Merino B[3], Clift MJD[2], Doak SH[2] and Catalán J[1,4]

[1]Finnish Institute of Occupational Health, Box 40, Työterveyslaitos, 00032 Helsinki, Finland

[2]In Vitro Toxicology Group, Faculty of Medicine, Health and Life Sciences, Institute of Life Sciences, Swansea University Medical School, Singleton Park, Swansea, SA2 8PP, Wales, UK

[3]TEMAS Solutions GmbH, 5212 Hausen, Switzerland

[4]Department of Anatomy Embryology and Genetics, University of Zaragoza, 50.013 Zaragoza, Spain

**Table S4.** Qualified *in vitro* studies on $TiO_2$ nanoparticles.

| Publication | Cell line | Assays | Nanomaterial | Dose range | Cellular uptake | Results |
|---|---|---|---|---|---|---|
| Catalán *et al.* 2012 [55] | Human lymphocytes | Chromosome aberration assay | $TiO_2$ anatase <25 nm | 6.25-300 µg/ml | Not assessed | Significant increase at 100 and 300 µg/ml at 48 h culture, with a significant dose-response. |
| Di Bucchianico *et al.* 2016 [52] | BEAS-2B | *In vitro* cytokinesis-blocked micronucleus assay<br><br>Flow-cytometric micronucleus assay | $TiO_2$ anatase NM-100 50–150 nm | 1-15 µg/mL | Quantified by flow cytometry (SSC) | Negative |
| | | | $TiO_2$ anatase NM-101 5–8 nm | 1-15 µg/mL | Quantified by flow cytometry (SSC) | Positive at 1 µg/mL, no significant dose-response. |
| | | | $TiO_2$ rutile NM-103 22-28 nm | 1-15 µg/mL | Quantified by flow cytometry (SSC) | Positive at 1-5 µg/mL, no significant dose-response. |
| Kazimirova *et al.* 2019 [51] | TK6 Human lymphocytes | *In vitro* cytokinesis-blocked micronucleus assay | $TiO_2$ anatase/rutile P25 AEROXIDE NM-105 15–30 nm | 3-75 µg/cm² (5-135 µg/mL) | Not assessed | Negative in TK6 and lymphocytes |
| Prasad *et al.* 2013 [44] | BEAS-2B | *In vitro* cytokinesis-blocked micronucleus assay | $TiO_2$ anatase/rutile P25 AEROXIDE 15–30 nm | 10-100 µg/ml | Confirmed at 24 h by flow cytometry and dark field microscopy | Significant increase at 20-100 µg/mL, together with significant dose-response, when dispersed in serum-containing medium. Negative in serum-free medium. |
| Shukla *et al.* 2011 [53] | A431 | *In vitro* cytokinesis-blocked micronucleus assay | $TiO_2$ anatase 50 nm | 0.008-80 µg/ml | Confirmed at 6 h by flow cytometry and TEM | Positive at 0.8-80 µg/mL |
| Stoccoro *et al.* 2016 [50] | Balb/3T3 | *In vitro* cytokinesis-blocked micronucleus assay | $TiO_2$ anatase/rutile P25 AEROXIDE 15–30 nm | 10-40 µg/cm² (32-128 µg/ml) | Confirmed at 48 h by TEM | Significant increase of MN at 10 µg/cm² |
| Zijno *et al.* 2015 [54] | Caco-2 | *In vitro* cytokinesis-blocked micronucleus assay | $TiO_2$ anatase 20-60 nm | 1-20 µg/cm² (6.4-128 µg/ml) | Not assessed | Negative |

Side scatter cytometry (SSC), Transmission electron microscopy (TEM)

**Table S5.** Qualified *in vivo* studies on $TiO_2$ nanoparticles.

| Publication | Exposure route & Organism | Assays | Nanomaterials | Dose range & treatment | Toxicokinetics | Results |
|---|---|---|---|---|---|---|
| Lindberg *et al.* 2012 [57] | Inhalation<br>Male C57BL/6J mice | Micronucleus assay (peripheral blood erythrocytes) | $TiO_2$<br>anatase/brookite<br>21 nm | 0.8-28.5 mg/m$^3$ 5-day repeated exposure, 4 h/day. Maximum dose limited by agglomeration. | Lung deposition was analyzed (ICP-MS). Bone marrow not assessed | Positive at 0.8 mg/m$^3$ |
| Relier *et al.* 2017 [56] | Intratracheal instillation<br>Male Sprague-Dawley rats | Micronucleus assay (peripheral blood erythrocytes) | $TiO_2$<br>anatase/rutile<br>P25 AEROXIDE (NM-105)<br>15–30 nm | 0.5-10 mg/kg 3x repeated treatment (4 d interval). Maximum dose limited by lung overload. | Material reaches lung and liver, but not observed in kidney or spleen (PIXE). Bone marrow not assessed | Positive on day 35 post-treatment at all tested doses |
| Shukla *et al.* 2014 [58] | Oral<br>Male Swiss albino mice | Comet assay (liver) | $TiO_2$<br>anatase<br>20–50 nm | 10-100 mg/kg bw<br>14-d repeated treatment. | Not assessed | Positive results measured by % of tail DNA in the standard and the Fpg-modified comet assays from 50 mg/kg |

Inductive couple plasma mass spectrometry (ICP-MS), particle induced X-ray emission (PIXE)

**Table S6.** Qualified *in vitro* studies on Ag nanoparticles.

| Publication | Cell line | Assays | Nanomaterial | Dose range | Cellular uptake | Results |
|---|---|---|---|---|---|---|
| Gábelová *et al.* 2016 [63] | L5178Y | Mouse lymphoma assay (MLA) | Ag NM-300 <20 nm | 0.3-100 µg/cm³ Limited by cytotoxicity | Not assessed | Negative |
| Li *et al.* 2012 [61] | TK6 | Flow-cytometric micronucleus (MN) assay | Ag 4-12 nm | 10-30 µg/ml Limited by cytotoxicity | Not assessed | Positive dose response, significant difference from control only at moderately cytotoxic doses. |
| Guo *et al.* 2016 [60] | TK6, L5718Y | Flow-cytometric micronucleus (MN) assay<br><br>Mouse lymphoma assay (MLA) | Ag, citrate-coated 20 nm, 50 nm and 100 nm | Between 2.5-400 µg/mL depending on the cytotoxicity of each material | Confirmed by TEM (confirmed by EDS for 50 nm citrate-coated Ag). Only assessed in L5718Y cells. | Size-dependent cytotoxicity and genotoxicity.<br>MN assay in TK6: Positive for 20-nm Ag particles, 50-nm particles negative. With 100-nm particles MN induction only at cytotoxic doses<br>MN assay in L5718Y: Positive for 20-nm particles. 50-nm particles positive only at a highly cytotoxic dose. For 100-nm particles equivocal result (no dose response).<br>MLA: Positive only at highly cytotoxic doses. |
| | | | Ag, PVP-coated 20 nm, 50 nm and 100 nm | Between 2.5-400 µg/mL depending on the cytotoxicity of each material | Confirmed by TEM. Only assessed in L5718Y cells. | Size-dependent cytotoxicity and genotoxicity.<br>MN assay in TK6: Positive for 20-nm Ag particles, 50-nm particles negative, for 100-nm particles positive only at highly cytotoxic dose.<br>MN assay in L5718Y: Significant MN induction only at cytotoxic doses.<br>MLA: Positive only at highly cytotoxic doses. |
| Nymark *et al.* 2013 [62] | BEAS-2B | *In vitro* cytokinesis-blocked micronucleus assay | Ag, PVP-coated 42.5 ± 14.5 nm | 2-48 µg/cm² (10-240 µg/ml) Limited by cytotoxicity | Not assessed | Negative |

Transmission electron microscopy (TEM), Energy-dispersive X-ray spectroscopy (EDS)

**Table S7.** Qualified *in vivo* studies on Ag nanoparticles.

| Publication | Exposure route & Organism | Assays | Nanomaterials | Dose range & treatment | Toxicokinetics | Results |
|---|---|---|---|---|---|---|
| Boudreau *et al.* 2016 [49] | Oral gavage Male and female Sprague-Dawley CD-23 rats | Micronucleus assay (peripheral blood erythrocytes) | Ag 10 nm, 75 nm, 110 nm | 9-36 mg/kg bw repeated daily for 13 w. Maximum dose justified by the dispersion method and gavage volume maximum. | Silver accumulation in blood and especially in bone marrow was minimal compared to other organs (ICP-MS). 110 nm Ag particles were not found in bone marrow. | Negative |
| Li *et al.* 2014 [64] | Intravenous Male B6C3F1 mice | Micronucleus assay (peripheral blood erythrocytes) | Ag, PVP-coated 5 nm | 0.5-20 mg/kg bw single treatment [a] | Not assessed | Negative |
| | | | Ag, PVP-coated 15-100 nm | 25 mg/kg bw single or 3x repeated treatment [a] | Material reached bone marrow and accumulated in liver (ICP-MS, TEM) | Negative |
| | | | Ag, silicon-coated 10-80 nm | 25 mg/kg bw single or 3x repeated treatment [a] | Material reached bone marrow and accumulated in liver (ICP-MS, TEM) | Negative |

Inductively coupled plasma mass spectrometry (ICP-MS), Transmission electron microscopy (TEM); [a] Unclear justification of dose range

**Table S8.** Qualified *in vitro* studies on metal-containing nanoparticles, excluding $TiO_2$ and Ag.

| Publication | Cell line | Assays | Nanomaterial | Dose range | Cellular uptake | Results |
|---|---|---|---|---|---|---|
| Uboldi *et al.* 2016 [1] | BEAS-2B | *In vitro* cytokinesis-blocked micronucleus assay | $Co_3O_4$ | 1.25-100 µg/ml Limited by cytotoxicity | Not assessed | Positive response at all tested doses, dose response not tested |
| Kumbıçak *et al.* 2014 [2] | BEAS-2B | *In vitro* cytokinesis-blocked micronucleus assay | Cu-Zn | 0.1-3.2 µg/ml | Confirmed by TEM | Positive at all tested doses. Significant dose-response. |
| Könczöl *et al.* 2011 [3] | A549 | *In vitro* cytokinesis-blocked micronucleus assay | $Fe_3O_4$ 20-60 nm | 1-100 µg/cm$^2$ | Confirmed by TEM | Positive at 10 and 100 µg/cm$^2$. Dose response not tested. |
| Di Bucchianico *et al.* 2018 [47] | BEAS-2B | *In vitro* cytokinesis-blocked micronucleus assay | Ni | 1-10 µg/mL | Confirmed by TEM and ICP-MS. | Significant increase of MN in binuclear cells at 5-10 µg/ml. Other types of chromosomal damage at all tested doses. Dose response not tested. |
| | | | NiO | 1-10 µg/mL | Confirmedby TEM and ICP-MS. | Significant increase of MN in binuclear cells at 5-10 µg/ml. Other types of chromosomal damage at all tested doses. Dose response not tested. |

| Publication | Cell line | Assays | Nanomaterial | Dose range | Cellular uptake | Results |
|---|---|---|---|---|---|---|
| Manshian *et al.* 2016 [4] | TK6 | *In vitro* cytokinesis-blocked micronucleus assay | Quantum dots, hexadecylamine -coated | 2.5-10 nM Limited by cytotoxicity | Could not be observed by TEM, confocal microscopy, and Imagestream flow cytometry at 18 h | Dose-dependent induction of MN at 2.5-10 nM |
| | | | Quantum dots, Amine-coated | 2.5-15 nM | Low levels of uptake observed by TEM, confocal microscopy, and Imagestream flow cytometry at 18 h | Positive at 2.5 nM, no dose response |
| | | | Quantum dots, Carboxyl-coated | 2.5-15 nM | Confirmed by TEM, confocal microscopy, and Imagestream flow cytometry at 18 h | Significant dose response, but none of the doses significantly differed from the negative control |
| Uboldi *et al.* 2019 [5] | BEAS-2B | *In vitro* cytokinesis-blocked micronucleus assay | W (pristine, hydrogenated or tritated) produced by plasma sputtering or laser ablation | 1-20 µg/ml | Not assessed | Positive response for all particle types and all doses, except plasma sputtering-produced pristine onesat 5 µg/ml. Dose response not tested. |
| Moche *et al.* 2014 [6] | L5178Y, Human lympho-cytes | *In vitro* cytokinesis-blocked micronucleus assay | WC-Co | 40-120 µg/ml (L5178Y cells) and 20-90 µg/ml (human lymphocytes) | Not assessed | Positive response at all tested doses in both cell types. Mainly aneugenic, but also some clastogenic damage. Dose response not assessed. |
| Senapati *et al.* 2015 [66] | THP-1 | *In vitro* cytokinesis-blocked micronucleus assay | ZnO | 0.5-20 µg/ml [b] | Confirmed by flow cytometry and TEM | Positive at 10-20 µg/mL, dose response not tested. |
| Zijno *et al.* 2015 [54] | Caco-2 | *In vitro* cytokinesis-blocked micronucleus assay | ZnO | 1-5 µg/cm$^2$ (6.4-32 µg/ml) Limited by cytotoxicity | Not assessed | Concluded as positive, but the conclusion should be disregarded as the results are significant only at highly cytotoxic doses and dose response was not tested. |

Transmission electron microscopy (TEM), Inductively coupled plasma mass spectrometry (ICP-MS)

**Table S9.** Qualified *in vivo* studies on metal-containing nanoparticles, excluding $TiO_2$ and Ag.

| Publication | Exposure route & Organism | Assays | Nano-materials | Dose range & treatment | Toxicokinetics | Results |
|---|---|---|---|---|---|---|
| Kumari *et al.* 2014 [7] | Oral gavage Female Wistar rats | Comet assay in blood leukocytes and liver. Micronucleus assay in blood and bone marrow erythrocytes Chromosome aberrations assay in bone marrow erythrocytes | $CeO_2$ | 100-1000 mg/ kg bw single treatment Limited by acute toxicity | Material found in liver, kidney, heart, spleen, brain, blood (ICP-OES). Bone marrow not assessed | Positive at 1000 mg/kg in all the assays |
| Singh *et al.* 2016 [8] | Oral gavage Male and Female Wistar rats | Micronucleus and chromosome aberrations assay in bone marrow erythrocytes Comet assay in blood cells and liver | $Cr_2O_3$ | 30-1000 mg/kg bw/day repeated treatment 28 d[a] | Dose-dependent biodistribution in peripheral blood, liver, kidneys, heart, brain, lung, and spleen (ICP-OES). Bone marrow not assessed | Positive result in all tests at 300 and 1000 mg/kg except at 300 mg/kg in the CA assay male group which was elevated but did not reach significance |
| Singh *et al.* 2013 [9] | Oral gavage Female Wistar rats | Chromosome aberrations assay in bone marrow erythrocytes Comet assay in blood cells | $Fe_2O_3$ | 500-2000 mg/kg bw single treatment | Accumulation in liver, spleen, kidney, heart and in small amounts in bone marrow (AAS) | Negative |
| Mangalampalli *et al.* 2017 [10] | Oral gavage Female Wistar rats | Chromosome aberrations assay in bone marrow erythrocytes Comet assay in blood cells and liver | MgO | 100-1000 mg/kg bw single treatment Limited by acute toxicity[a] | Dose dependent bioaccumulation of Mg in liver, kidneys, blood, spleen, heart, and brain (ICP-OES). Bone marrow not assessed. | Comet and CA positive at 500-1000 mg/kg. Cytotoxicity observed at 1000 mg/kg in CA assay. |
| Singh *et al.* 2013 [11] | Oral gavage Male and Female Wistar rats | Micronucleus and chromosome aberrations assay in bone marrow erythrocytes Comet assay in blood cells | $MnO_2$ | 30-1000 mg/kg bw/day 28-day repeated treatment. [a] | Dose-pedendent biodistribution in PB, liver, kidneys, heart, brain, lung, and spleen (ICP-MS). Bone marrow not assessed | Positive result in all tests at 300 and 1000 mg/kg |
| Dumala *et al.* 2017 [67] | Oral gavage Female Wistar rats | Micronucleus and chromosome aberrations assay in bone marrow erythrocytes Comet assay in blood cells, kidney and liver | NiO | 125-500 mg/kg bw single treatment [a] | Material found in PB, brain, heart, liver, kidneys, and spleen (ICP-OES). Bone marrow not assessed | Positive results in comet assay starting from 250 mg/kg (PB, kidney) and 500 mg/kg (liver), and in both MN and CA assay (BM) starting from 250 mg/kg. |

| Publication | Exposure route & Organism | Assays | Nano-materials | Dose range & treatment | Toxicokinetics | Results |
|---|---|---|---|---|---|---|
| Chinde *et al.* 2017 [68] | Oral gavage Female Wistar rats | Micronucleus assay in blood and bone marrow erythrocytes<br>Chromosome aberrations assays in blood erythrocytes<br>Comet assay in blood cells and liver | $WO_3$ | 100-1000 mg/kg bw, single treatment [a] | Accumulation in all tissues except bone marrow, which was not assessed (ICP-OES) | All tests positive at 1000 mg/kg |
| Chinde *et al.* 2017 [69] | Oral gavage Male and Female Wistar rats | Micronucleus assay in bone marrow erythrocytes<br>Comet assay in blood cells and liver | $WO_3$ | 250-1000 mg/kg bw repeated daily for 28 d [ab] | Accumulation in all tissues except bone marrow, which was not assessed (ICP-OES) | All tests positive at 1000 mg/kg |
| Panyala *et al.* 2017 [70] | Oral gavage Female Wistar rats | Micronucleus assay in blood and bone marrow erythrocytes<br>Comet assay in blood cells and liver | $Y_2O_3$ | 250-1000 mg/kg bw single treatment up to a low toxicity dose | Accumulation in liver, kidneys, spleen, lung, heart, brain and blood (ICP-OES). Bone marrow not assessed | All tests positive at 1000 mg/kg |
| Panyala *et al.* 2019 [71] | Oral gavage Male and Female Wistar rats | Micronucleus assay in blood and bone marrow erythrocytes<br>Comet assay in blood cells and liver | $Y_2O_3$ | 30-480 mg/kg bw repeated daily for 28 d up to a low toxicity dose | Accumulation in liver, kidney, blood, intestine, lungs, spleen, heart, and brain (ICP-OES). Bone marrow not assessed | All tests positive at 120-480 mg/kg |

Inductively Coupled Plasma Optical Emission Spectrometry (ICP-OES), Atomic absorption spectroscopy (ASS), [a] Unclear justification of dose range [b] Dose range based on an acute toxicity study, but data not shown or no reference

**Table S10.** Qualified *in vitro* publications related to nanofibers.

| Publication | Cell line | Assays | Nanomaterial(s) | Dose range | Cellular uptake | Results |
|---|---|---|---|---|---|---|
| Catalán *et al.* 2016 [76] | BEAS-2B | *In vitro* cytokinesis-blocked micronucleus assay | MWCNT-S | 0, 2.5, 5, 10, 20, 50, 100 μg/cm². | Not assessed | Negative. |
| Louro *et al.* 2016 [77] | A549<br>BEAS-2B | *In vitro* cytokinesis-blocked micronucleus assay | NM400, NM401, NM402, NM403 | 0, 25, 50, 75, 100, 125, 150 μg/cm². | Not assessed | Significant response for NM401, NM402 only in A549 cells at highest concentration. |
| Manshian *et al.* 2013 [75] | BEAS-2B<br>MCL-5 | *In vitro* cytokinesis-blocked micronucleus assay<br>*Hprt* forward mutation assay (MCL-5 only) | Three types of SWCNTs:<br><br>400-800nm<br>1-3μm<br>5-30μm | 0, 1, 5, 10, 15, 20, 25, 50, 100 μg/ml | Confirmed in both cell lines | *In vitro* CBMN assay: 400-800nm sample positive at all test doses both cell types.<br>*Hprt* forward mutation assay: Positive response with the 1-3μm SWCNTs at 25, 50, 100μg/ml in MCL-5 cells. |
| Tavares *et al.* 2014 [78] | Blood-derived human lymphocytes | *In vitro* cytokinesis-blocked micronucleus assay | MWCNTs: (NM-400, NM-401, NM-402, NM-403, NRCWE-006 and NRCWE-007) | 0, 5, 10, 15, 45, 60, 100, 105, 125, 250 μg/ml (material-specific) | Not assessed | Significant increase in binucleated cells with micronuclei were detected after exposure to one dose of NM-402, 15μg/ml, to NRCWE-006 at 2.5 and 15μg/ml and to all doses of NM-403 except at 125μg/ml. The remaining MWCNT did not induce micronuclei in human lymphocytes. |
| Vales *et al.* 2016 [12] | BEAS-2B | *In vitro* cytokinesis-blocked micronucleus assay | MWCNT, NM403 | 0, 1, 20, 20 μg/ml | Not assessed | Positive at 10μg/ml (24-hour exposure), also positive at 20μg/ml (but only following 1-week and 3-week exposures). |
| Di Giorgio *et al.* 2011 [13] | RAW 264.7 macrophages | *In vitro* cytokinesis-blocked micronucleus assay | SWCNT & MWCNT | 0, 1, 3, 10, 50μg/ml | TEM utilised; uptake confirmed | Positive at all doses in the CBMN assay. |
| Catalán *et al.* 2012 [55] | Human-derived lymphocytes | *In vitro* chromosomal aberration assay | SWCNT & MWCNT | 6.25-300μg/ml | Not assessed | SWCNT induced a positive result but only at very high concentrations of 300μg/ml and after 48- and 72-hour exposure.<br>MWCNT induced a positive result at 50μg/ml following 24-hour exposure. At 48- and 72-hour exposures, both 50 and 300μg/ml induced a positive response. |

Multiwalled carbon nanotubes (MWCNT); single walled carbon nanotubes (SWCNT); Transmission electron microscopy (TEM)

**Table S11.** Qualified *in vivo* publications related to nanofibers.

| Publication | Organism & exposure route | Assays | Nano-materials | Dose range | Toxicokinetics | Results |
|---|---|---|---|---|---|---|
| Catalán *et al.* 2017 [79] | Female C57Bl/6 mice, 7–8 weeks old; ~20g per mouse<br><br>Pharyngeal aspiration | In vivo comet assay<br><br>Automated micronucleus assay in bone marrow erythrocytes | Nano fibrillated cellulose (NFC) | 0, 10, 40, 80, 200 µg/mouse & controls | Dose-dependent accumulation. Found within bronchi and alveoli, also inside macrophages.<br>Confirmed by histological examination and TEM imaging. | Comet assay: Significant %DNA in tail in lung cells<br><br>Negative response in micronucleus |
| Catalán *et al.* 2016 [76] | Female C57Bl/6 mice, 7–8 weeks old<br><br>Pharyngeal aspiration<br>Inhalation exposure | In vivo comet assay<br><br>Automated micronucleus assay in bone marrow erythrocytes<br><br>Micronucleus assay in tail blood erythrocytes | MWCNTs | Material-dependent, total dose range:<br>0, 10, 20, 30, 40, 50, 100, 150, 200 µg/mouse & controls | Dose-dependent accumulation of both types of MWCNTs following pharyngeal aspiration mainly in the bronchia and to lesser extent the alveoli.<br><br>The inhalation exposure revealed accumulation mainly in alveolar tissue | Comet assay: significant result at highest dose following pharyngeal aspiration.<br>Significant response in inhalation exposures.<br><br>Negative response in bone marrow erythrocytes. |
| Ema *et al.* 2012 [14] | Seven-week-old Crlj: CD1 (ICR) mice<br><br>Oral gavage | In vivo erythrocyte micronucleus assay | MWCNTs | 0, 5, 10, 20 mg/kg | Toxicokinetics not assessed | Negative |
| Naya *et al.* 2011 [15] | Six-week-old Crlj: CD1 (ICR) mice | In vivo bone marrow micronucleus test | SWCNTs | 0, 60, 200 mg/kg & controls | Toxicokinetics not investigated | Negative |
| Pothmann *et al.* 2015 [80] | Male and female Rats, RccHan©: WIST(SPF). Male BW: 291-347g and 243-296g 176 to 214g. Female BW: 135 to 228g<br><br>Nose-only inhalation | In vivo micronucleus assay<br><br>In vivo comet assay | Graphistrength C100 (MWCNT) | 0, 0.05, 0.25, 5 & controls | Histological examination revealed agglomerates within alveolar and tissue macrophages. | Negative response in polychromatic erythrocyte micronucleus frequencies.<br><br>Negative in comet assay. |
| Christophersen *et al.* 2016 [81] | Female C57BL/6-Ntac mice<br><br>Oral and pulmonary exposure | In vivo comet assay | MWCNT-7 | 0, 40, 400 µg | Toxicokinetics not investigated | Negative |

| Publication | Organism & exposure route | Assays | Nano-materials | Dose range | Toxicokinetics | Results |
|---|---|---|---|---|---|---|
| | Oral gavage<br>Intratracheal instillation | | | | | |
| Honda *et al.* 2017 [82] | 9-week-old male F344/DuCrlCrlj male rats<br><br>A single IT instillation | In vivo comet assay | SWCNTs | 0, 0.3-1 mg/kg | Toxicokinetics confirmed using histological examination and TEM<br><br>Deposition in the alveolus, short fibres found in the mediastinum lymph nodes. | Negative |
| Jacobsen *et al.* 2009 [16] | Female wild-type C57BL/6 (C57) and C57BL/6-Apoe$^{tm1}$ (ApoE$^{-/-}$) mice aged 4–6 weeks<br><br>A single IT instillation or single inhalation | In vivo comet | SWCNTs | 0-54 µg | Toxicokinetics not investigated | Positive response when measuring tail length. When measuring %DNA in tail levels were elevated but not significant. |
| Patlolla *et al.* 2010 [84] | adult male Swiss-Webster mice (5-7 weeks of age, with average body weight (BW) of 30 ± 2g)<br><br>intraperitoneal | Chromosome aberration assay.<br>Micronucleus<br>Comet | MWCNT | 0.25, 0.5, 0.75 mg/kg | Toxicokinetics not investigated | Top two doses positive in comet.<br>Positive for micronuclei induction.<br>Positive for CA assay |
| Patlolla *et al.* 2016 [85] | adult male Swiss-Webster mice (6-8 weeks of age, with average body weight (BW) of 30 ± 2g)<br><br>intraperitoneal | Chromosome aberration assay.<br>Micronucleus<br>Comet | SWCNT | 0.25, 0.5, 0.75 mg/kg | Toxicokinetics not investigated | Positive for CA assay at top two doses.<br>Positive for Mn at top two doses.<br>Positive in comet at top two doses. |
| Poulsen *et al.* 2016 [17] | Female mice C57BL/6J BomTac aged 6–7 weeks (19 ± 1.5 g)<br><br>Intratracheal instillation | In vivo comet assay | MWCNT | 0, 6, 18 and 54 µg/mouse | Deposits confirmed in alveolar macrophages. | Positive responses observed. |

| Publication | Organism & exposure route | Assays | Nano-materials | Dose range | Toxicokinetics | Results |
|---|---|---|---|---|---|---|
| Rahman *et al.* 2017 [18] | Adult 12-week-old female Muta™Mouse<br><br>Intratracheal instillation | In vivo comet | MWCNT | Designated low or high dose:<br>Low: 26-36 µg/mouse<br>High: 78-109 µg/mouse | Histopathology showed significant presence of MWCNTs 90 days after exposure, mainly in the fibrotic foci. | Negative except for high doses of NM401 which were positive. |

Multiwalled carbon nanotubes (MWCNT); single walled carbon nanotubes (SWCNT)

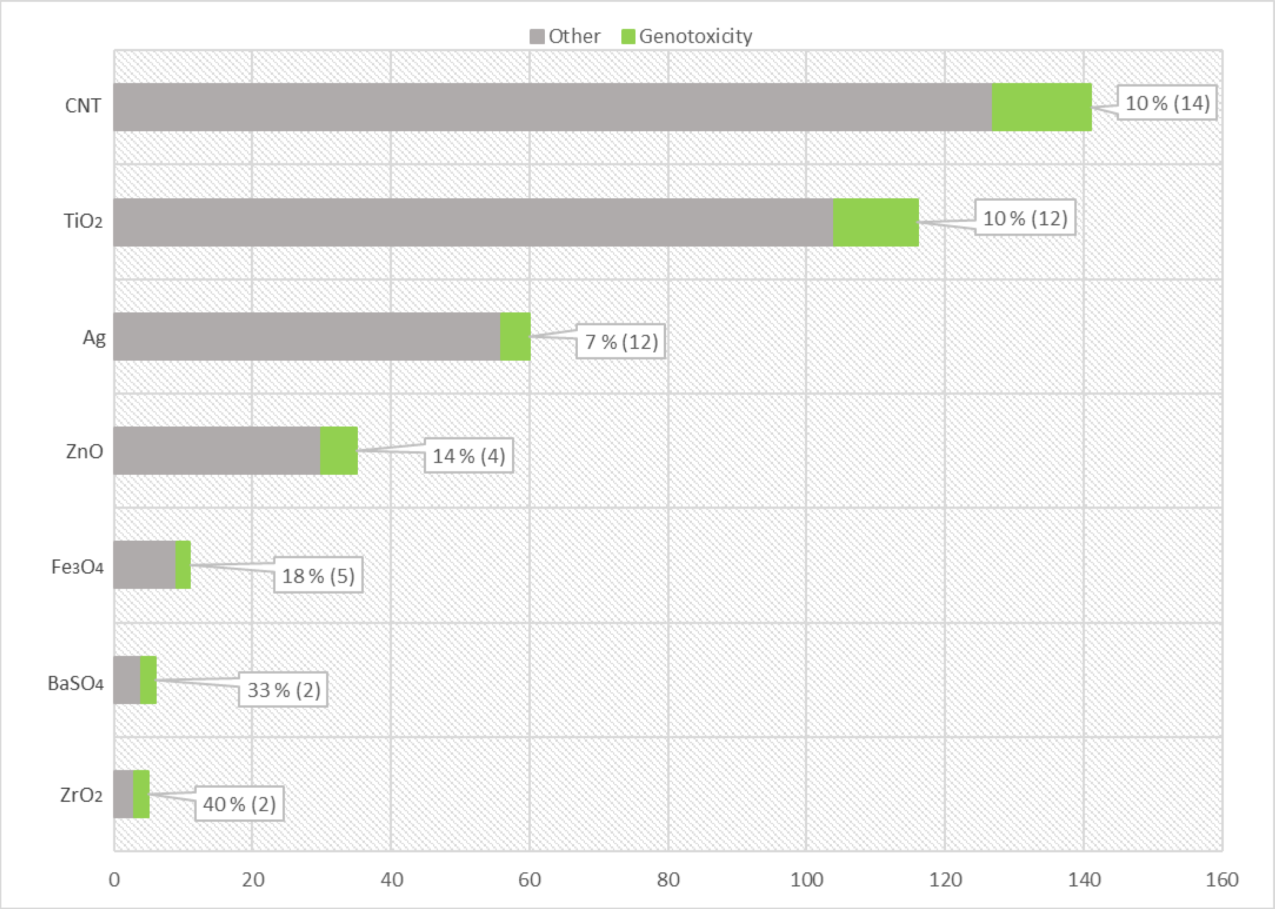


**Figure S1.** Percentage and total amount (in parenthesis) of genotoxicity data (green portion of the bar) compared to the total toxicity data entries (grey portion of the bars) collected from the NanoInformaTIX instance per nanomaterial. The X-axis indicates the number of entries.

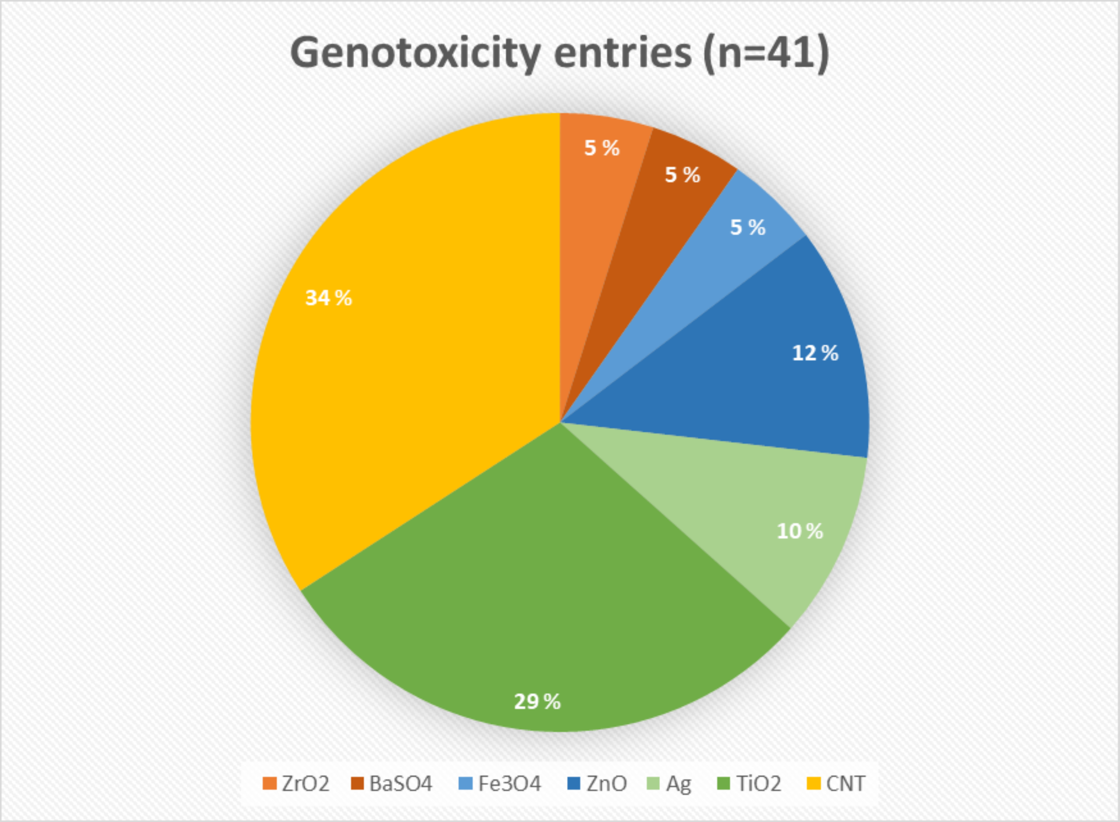


**Figure S2.** Nanomaterials for which available genotoxicity information was identified in the NanoInformaTIX instance. Data are expressed as the percentage of entries related to genotoxicity per each material compared to the total genotoxicity-related entries.

**Table S12.** Different nanoforms identified under the general nanomaterial names in the search performed in the NanoInformaTIX instance.

| Material | Name | Description | Supplier (Institution or name of the project) |
|---|---|---|---|
| **Carbon nanotubes** | JRCNM04000a | MWCNT 13.6 nm (diameter) | JRC - IHCP,Fraunhofer |
| | JRCNM04001a | MWCNT 64.2 nm (diameter) | JRC – IHCP |
| | JRCNM04002a | MWCNT 12.7 nm (diameter) | JRC - IHCP |
| | NRCWE-006 (Mitsui) | MWCNT 49 nm (diameter) | NanoGenotox |
| | JRCNM04003a | MWCNT 12 nm (diameter) | JRC - IHCP |
| | C_1.1_NT | MWCNT Aspect ratio 14.6 | Sanowork |
| | NM-411 | SWCNT 2 nm (diameter) | Fraunhofer,JRC – IHCP |
| | NRCWE-007 | MWCNT 74 nm (diameter) | JRC - IHCP |
| **TiO2, coating, primary particle size, (structure)** | TIO2_1.1_NF | Aspect ratio 0.0345<br>Diameter 0.34 um length 9.9 um | Sanowork |
| | TIO2_6_SOL | Mean particle size 4 nm | Sanowork |
| | TIO2_36.2_CIT_SOL | Mean particle size 5 nm | Sanowork |
| | JRCNM01005a | Mean particle size 3.4 nm | JRC - IHCP |
| | NRCWE-002 | Rutile 10 nm positive charge | MARINA |
| | NRCWE-003 | Rutile 10 nm negative charge | ENPRA |
| | NRCWE-001 | Rutile 10 nm | NanoAmor |
| | NRCWE-004 | Rutile 94 nm | NanoBond |
| | JRCNM01001a | Anatase 6 nm | JRC - IHCP,Fraunhofer |
| | JRCNM01000a | 50-150 nm | JRC - IHCP,Fraunhofer |
| | JRCNM01002a | Anatase 21-22 nm | JRC – IHCP |
| | JRCNM01003a | Rutile 24.7 nm | JRC – IHCP |
| | JRCNM01004a | Rutile 125 nm | JRC |
| | JRCNM62002a | Rutile 21.nm | JRC – IHCP |
| **ZnO** | JRCNM01100a | 5-6nm (diameter) | JRC – IHCP |
| | JRCNM01101a | 73 nm (diameter) | JRC - IHCP,Fraunhofer |
| **BaSO4** | NM-220 | 25 nm (diameter) | Fraunhofer,JRC - IHCP,IIT |
| **Ag** | NM-300K | 16.7 nm (diameter) | Fraunhofer,JRC – IHCP |
| | AG_1_SOL | 24 nm (diameter) | Sanowork |
| | AG_31_SOL_UF | - | Sanowork |
| **Fe3O4** | PL-A-FE3O4 | 8 nm (diameter) | NanoTest |
| | PL-M-FE3O4 | 8 nm (diameter) | Nanotest |
| **ZrO2** | ZRO2_2_SOL | - | Sanowork |

| | | | |
|---|---|---|---|
| | ZRO2_10_CIT_SOL | - | Sanowork |
| | ZRO2_13.2_CIT_SOL | 10 nm | Sanowork |
| | ZRO2_7_SIL_SOL | 21 nm | Sanowork |

**Table S13.** List of different names associated to a genotoxicity assay as found in the NanoInformaTIX instance.

| Assay Name in eNanomapper[a] | Assay | Link to a SOP[b] |
|---|---|---|
| **Comet** | Standard Comet assay with adaptations to NM | Open access link to a SOP |
| **Comet (ox DNA damage)** | FPG modified Comet | Protocol not provided |
| **Comet (primary DNA damage)** | Standard Comet | Protocol not provided |
| **NET-Fpg** | FPG modified Comet | Link to a SOP provided but not working |
| **Comet-SB** | Standard Comet | Link to a SOP provided but not working |
| **Cell Transformation Assay** | Cell transformation assay (CTA) | A reference to a CTA method is reported but there is no link to a SOP. |
| **DNA Strand breaks** | Standard Comet | A reference to the Comet method is indicated but there is no link to a SOP |
| ***In vitro* micronucleus** | Standard in vitro micronucleus assay with adaptations to NM | Open access link to a SOP |
| **Genotoxicity** | FPG modified Comet | Open access link to a SOP corresponding to the protocol of Azqueta et al (2014) with minor modifications |
| | High Content Analysis (H2AX phospho S139) | No link. Only a reference to NanoReg D5.07 |
| | High Content Analysis (p53 phospho s15) | No link. Only a reference to NanoReg D5.07 |
| | High Content Analysis (ATM phospho S1981) | No link. Only a reference to NanoReg D5.07 |
| | High Content Analysis (ATM phospho S1981) | No link. Reference to SOP ATM phospho S1981 |
| ***in vivo* comet assay** | OECD TG 474: Mammalian Erythrocyte Micronucleus Test[c] | Open access link to a SOP |
| **micronucleus** | Standard in vitro micronucleus assay with adaptations to NM | NanoReg D5.07 open access |
| **mouse lymphoma L5178Y/TK+/- assay** | Mouse lymphoma assay | No link. Reference to method MLA-TK |
| **genetic toxicity *in vivo*** | In vivo micronucleus assay | NANoREF D4.15 protocol for inhalation exposure and choice of biological relevant endpoints |

[a]As reported by the laboratory that enters the information
[b]SOP: Standard Operation Procedure
[c]The name of the entry refers to the comet assay, although the data included corresponds to the micronucleus assay

**Table S14.** Questions addressed regarding the genotoxicity data found in the NanoInformaTIX instance to fulfil the S score criteria.

| Question | Answer |
|---|---|
| ***Was the test substance identified?*** | Yes, a substance identifier could be found under some or all of the following; IUC substance name, IUC substance UUID, JRC name, UIC Public name, chemical name, UIPAC name, UUID. |
| ***Is information on the source/origin of the substance given?*** | Yes, this information was sometimes provided through a code from question one. |
| ***Is purity (concentration) of the substance given?*** | This information is provided under P-CHEM analytical methods. |
| ***Is endotoxin content of the substance given?*** | No, this information was not found. |
| ***Were impurities stated?*** | Yes, this information is provided under P-CHEM analytical methods. |
| ***Was the substance concentration measured in the exposure medium?*** | Yes, and the medium is indicated under P-CHEM analytical methods. |
| ***When the substance is a nanoparticle (NP), were protocols of dispersion and characterization in the exposure medium identified? or, were protocols of preparation of exposure medium stated?*** | Dispersion protocols were generally included. The composition of the exposure medium was generally provided. |
| ***Was the stability of the substance concentration measured during the exposure period?*** | The physico-chemical information provided in the database generally relates to one particular time point. |
| ***Are doses administered or concentrations in exposure media given?*** | Yes, exposed concentrations were reported |
| ***Was the type of test medium or vehicle used stated?*** | Yes, the composition of the media was described. |
| ***Were physico-chemical properties of the NMs provided?*** | Yes, materials were quite well-characterized |

**Table S15.** Questions addressed regarding the genotoxicity data found in the NanoInformaTIX instance to fulfil the *in vitro* K score criteria

| Question | Answer |
|---|---|
| ***Was the cell model or organism given?*** | Yes, information on the cell type was provided by name of cell type under the column labelled "protocol" |
| ***Was information given on the source/origin of the test system?*** | This information was generally not found. |
| ***Were necessary information on test system properties, and on conditions of cultivation and maintenance given?*** | Yes, this information was provided under the columns termed "protocol" within this column links to standard operating procedures (SOPs) were generally found. |
| ***Was the method of administration given (see explanations for details)?*** | Yes, this information was provided under the column termed "protocol" and/or "concentration" and/or "treatment". |
| ***Were duration of exposure as well as time-points of observations explained?*** | Duration of exposure was provided under the column termed "protocol". However, no explanations were provided since the input follows a database format, populated with single entries. |
| ***Were negative and positive controls included (where and when needed)?*** | Yes, this information was provided under the column termed "protocol", which includes a reference to positive and negative controls used in the assay |
| ***Was the number of replicates (or complete repetitions of experiment) given?*** | Yes, but in the form of independent entries. An independent entry was generally considered as a biological replicate. |
| ***Were the study endpoint(s) and their method(s) of determination clearly described?*** | Yes, through a link to the SOP under the column termed "protocol". |
| ***Had the results been analysed using statistical methods?*** | Average and standard deviations values are generally provided under the column termed "results", |

**Table S16.** Questions addressed regarding the genotoxicity data found in the NanoInformaTIX instance to fulfil the in vivo K score criteria

| Question | Answer |
|---|---|
| ***Was the species given?*** | Yes, it was provided under the column termed “protocol”. |
| ***Was the sex of the test organism given?*** | This information was not found though the followed OECD test guideline is included. |
| ***Was information given on the strain of test animals?*** | This information was not always found. |
| ***Was age or body weight of the test organisms at the start of the study given?*** | This information was not found. |
| ***Was information given on the housing or feeding conditions?*** | Yes, this information was provided. |
| ***Was the administration route given?*** | Yes, this was found under the column labelled with the OECD protocol |
| ***Were frequency and duration of exposure as well as time-points of observations explained?*** | Only exposure time was found. |
| ***Were negative (where required) and positive controls (where required) included?*** | Information was not found in the database but links to protocols were included. |
| ***Was the number of animals per group given?*** | This information was not found |
| ***Were sufficient details of the administration scheme given to judge the study?*** | Not much information is provided on the database but links to the protocols are found. |
| ***Had the study methods been described?*** | This information was provided through a link to the protocol |
| ***Had the results been analysed using statistical methods?*** | Average and standard deviations values are generally provided under the column termed “results”. |

# References

(as numbered in the main manuscript)

55. Catalán J, Järventaus H, Vippola M, Savolainen K, Norppa H. Induction of chromosomal aberrations by carbon nanotubes and titanium dioxide nanoparticles in human lymphocytes in vitro. Nanotoxicology. 2012;6(8):825-36, doi:10.3109/17435390.2011.625130.

52. Di Bucchianico S, Cappellini F, Le Bihanic F, Zhang Y, Dreij K, Karlsson HL. Genotoxicity of TiO2 nanoparticles assessed by mini-gel comet assay and micronucleus scoring with flow cytometry. Mutagenesis. 2016;32(1):127-37, doi:10.1093/mutage/gew030.

51. Kazimirova A, Baranokova M, Staruchova M, Drlickova M, Volkovova K, Dusinska M. Titanium dioxide nanoparticles tested for genotoxicity with the comet and micronucleus assays in vitro, ex vivo and in vivo. Mutation Research/Genetic Toxicology and Environmental Mutagenesis. 2019;843:57-65, doi:https://doi.org/10.1016/j.mrgentox.2019.05.001.

44. Prasad RY, Wallace K, Daniel KM, Tennant AH, Zucker RM, Strickland J, et al. Effect of Treatment Media on the Agglomeration of Titanium Dioxide Nanoparticles: Impact on Genotoxicity, Cellular Interaction, and Cell Cycle. ACS Nano. 2013;7(3):1929-42, doi:10.1021/nn302280n.

53. Shukla RK, Sharma V, Pandey AK, Singh S, Sultana S, Dhawan A. ROS-mediated genotoxicity induced by titanium dioxide nanoparticles in human epidermal cells. Toxicol In Vitro. 2011;25(1):231-41, doi:10.1016/j.tiv.2010.11.008.

50. Stoccoro A, Di Bucchianico S, Uboldi C, Coppedè F, Ponti J, Placidi C, et al. A panel of in vitro tests to evaluate genotoxic and morphological neoplastic transformation potential on Balb/3T3 cells by pristine and remediated titania and zirconia nanoparticles. Mutagenesis. 2016;31(5):511-29, doi:10.1093/mutage/gew015.

54. Zijno A, De Angelis I, De Berardis B, Andreoli C, Russo MT, Pietraforte D, et al. Different mechanisms are involved in oxidative DNA damage and genotoxicity induction by ZnO and TiO2 nanoparticles in human colon carcinoma cells. Toxicol In Vitro. 2015;29(7):1503-12, doi:10.1016/j.tiv.2015.06.009.

57. Lindberg HK, Falck GC, Catalán J, Koivisto AJ, Suhonen S, Järventaus H, et al. Genotoxicity of inhaled nanosized TiO(2) in mice. Mutat Res. 2012;745(1-2):58-64, doi:10.1016/j.mrgentox.2011.10.011.

56. Relier C, Dubreuil M, Lozano Garcìa O, Cordelli E, Mejia J, Eleuteri P, et al. Study of TiO2 P25 Nanoparticles Genotoxicity on Lung, Blood, and Liver Cells in Lung Overload and Non-Overload Conditions After Repeated Respiratory Exposure in Rats. Toxicological Sciences. 2017;156(2):527-37, doi:10.1093/toxsci/kfx006.

58. Shukla RK, Kumar A, Vallabani NV, Pandey AK, Dhawan A. Titanium dioxide nanoparticle-induced oxidative stress triggers DNA damage and hepatic injury in mice. Nanomedicine (Lond). 2014;9(9):1423-34, doi:10.2217/nnm.13.100.

63. Gábelová A, El Yamani N, Alonso TI, Buliaková B, Srančíková A, Bábelová A, et al. Fibrous shape underlies the mutagenic and carcinogenic potential of nanosilver while surface chemistry affects the biosafety of iron oxide nanoparticles. Mutagenesis. 2016;32(1):193-202, doi:10.1093/mutage/gew045.

61. Li Y, Chen DH, Yan J, Chen Y, Mittelstaedt RA, Zhang Y, et al. Genotoxicity of silver nanoparticles evaluated using the Ames test and in vitro micronucleus assay. Mutat Res. 2012;745(1-2):4-10, doi:10.1016/j.mrgentox.2011.11.010.

60. Guo X, Li Y, Yan J, Ingle T, Jones MY, Mei N, et al. Size- and coating-dependent cytotoxicity and genotoxicity of silver nanoparticles evaluated using in vitro standard assays. Nanotoxicology. 2016;10(9):1373-84, doi:10.1080/17435390.2016.1214764.

62. Nymark P, Catalán J, Suhonen S, Järventaus H, Birkedal R, Clausen PA, et al. Genotoxicity of polyvinylpyrrolidone-coated silver nanoparticles in BEAS 2B cells. Toxicology. 2013;313(1):38-48, doi:10.1016/j.tox.2012.09.014.

49. Boudreau MD, Imam MS, Paredes AM, Bryant MS, Cunningham CK, Felton RP, et al. Differential Effects of Silver Nanoparticles and Silver Ions on Tissue Accumulation, Distribution, and Toxicity in the Sprague Dawley Rat Following Daily Oral Gavage Administration for 13 Weeks. Toxicological Sciences. 2016;150(1):131-60, doi:10.1093/toxsci/kfv318.

64. Li Y, Bhalli JA, Ding W, Yan J, Pearce MG, Sadiq R, et al. Cytotoxicity and genotoxicity assessment of silver nanoparticles in mouse. Nanotoxicology. 2014;8(sup1):36-45, doi:10.3109/17435390.2013.855827.

47. Di Bucchianico S, Gliga AR, Åkerlund E, Skoglund S, Wallinder IO, Fadeel B, et al. Calcium-dependent cyto- and genotoxicity of nickel metal and nickel oxide nanoparticles in human lung cells. Particle and Fibre Toxicology. 2018;15(1):32, doi:10.1186/s12989-018-0268-y.

66. Senapati VA, Kumar A, Gupta GS, Pandey AK, Dhawan A. ZnO nanoparticles induced inflammatory response and genotoxicity in human blood cells: A mechanistic approach. Food and Chemical Toxicology. 2015;85:61-70, doi:https://doi.org/10.1016/j.fct.2015.06.018.

67. Dumala N, Mangalampalli B, Chinde S, Kumari SI, Mahoob M, Rahman MF, et al. Genotoxicity study of nickel oxide nanoparticles in female Wistar rats after acute oral exposure. Mutagenesis. 2017;32(4):417-27, doi:10.1093/mutage/gex007.

68. Chinde S, Dumala N, Rahman MF, Kamal SSK, Kumari SI, Mahboob M, et al. Toxicological assessment of tungsten oxide nanoparticles in rats after acute oral exposure. Environ Sci Pollut Res Int. 2017;24(15):13576-93, doi:10.1007/s11356-017-8892-x.

69. Chinde S, Grover P. Toxicological assessment of nano and micron-sized tungsten oxide after 28days repeated oral administration to Wistar rats. Mutat Res Genet Toxicol Environ Mutagen. 2017;819:1-13, doi:10.1016/j.mrgentox.2017.05.003.

70. Panyala A, Chinde S, Kumari SI, Grover P. Assessment of genotoxicity and biodistribution of nano- and micron-sized yttrium oxide in rats after acute oral treatment. J Appl Toxicol. 2017;37(12):1379-95, doi:10.1002/jat.3505.

71. Panyala A, Chinde S, Kumari SI, Rahman MF, Mahboob M, Kumar JM, et al. Comparative study of toxicological assessment of yttrium oxide nano- and microparticles in Wistar rats after 28 days of repeated oral administration. Mutagenesis. 2019;34(2):181-201, doi:10.1093/mutage/gey044.

76. Catalán J, Siivola KM, Nymark P, Lindberg H, Suhonen S, Järventaus H, et al. In vitro and in vivo genotoxic effects of straight versus tangled multi-walled carbon nanotubes. Nanotoxicology. 2016;10(6):794-806, doi:10.3109/17435390.2015.1132345.

77. Louro H, Pinhão M, Santos J, Tavares A, Vital N, Silva MJ. Evaluation of the cytotoxic and genotoxic effects of benchmark multi-walled carbon nanotubes in relation to their physicochemical properties. Toxicol Lett. 2016;262:123-34, doi:10.1016/j.toxlet.2016.09.016.

75. Manshian BB, Jenkins GJ, Williams PM, Wright C, Barron AR, Brown AP, et al. Single-walled carbon nanotubes: differential genotoxic potential associated with physico-chemical properties. Nanotoxicology. 2013;7(2):144-56, doi:10.3109/17435390.2011.647928.

78. Tavares AM, Louro H, Antunes S, Quarré S, Simar S, De Temmerman PJ, et al. Genotoxicity evaluation of nanosized titanium dioxide, synthetic amorphous silica and multi-walled carbon nanotubes in human lymphocytes. Toxicol In Vitro. 2014;28(1):60-9, doi:10.1016/j.tiv.2013.06.009.

79. Catalán J, Rydman E, Aimonen K, Hannukainen KS, Suhonen S, Vanhala E, et al. Genotoxic and inflammatory effects of nanofibrillated cellulose in murine lungs. Mutagenesis. 2017;32(1):23-31, doi:10.1093/mutage/gew035.

80. Pothmann D, Simar S, Schuler D, Dony E, Gaering S, Le Net JL, et al. Lung inflammation and lack of genotoxicity in the comet and micronucleus assays of industrial multiwalled carbon nanotubes Graphistrength(©) C100 after a 90-day nose-only inhalation exposure of rats. Part Fibre Toxicol. 2015;12:21, doi:10.1186/s12989-015-0096-2.

81. Christophersen DV, Jacobsen NR, Andersen MH, Connell SP, Barfod KK, Thomsen MB, et al. Cardiovascular health effects of oral and pulmonary exposure to multi-walled carbon nanotubes in ApoE-deficient mice. Toxicology. 2016;371:29-40, doi:10.1016/j.tox.2016.10.003.

82. Honda K, Naya M, Takehara H, Kataura H, Fujita K, Ema M. A 104-week pulmonary toxicity assessment of long and short single-wall carbon nanotubes after a single intratracheal instillation in rats. Inhal Toxicol. 2017;29(11):471-82, doi:10.1080/08958378.2017.1394930.

84. Patlolla AK, Hussain SM, Schlager JJ, Patlolla S, Tchounwou PB. Comparative study of the clastogenicity of functionalized and nonfunctionalized multiwalled carbon nanotubes in bone marrow cells of Swiss-Webster mice. Environ Toxicol. 2010;25(6):608-21, doi:10.1002/tox.20621.

85. Patlolla AK, Patra PK, Flountan M, Tchounwou PB. Cytogenetic evaluation of functionalized single-walled carbon nanotube in mice bone marrow cells. Environ Toxicol. 2016;31(9):1091-102, doi:10.1002/tox.22118.

(not included in the main manuscript)

1. Uboldi C, Orsière T, Darolles C, Aloin V, Tassistro V, George I, et al. Poorly soluble cobalt oxide particles trigger genotoxicity via multiple pathways. Particle and Fibre Toxicology. 2016;13(1):5, doi:10.1186/s12989-016-0118-8.

2. Kumbıçak U, Cavaş T, Cinkılıç N, Kumbıçak Z, Vatan O, Yılmaz D. Evaluation of in vitro cytotoxicity and genotoxicity of copper-zinc alloy nanoparticles in human lung epithelial cells. Food Chem Toxicol. 2014;73:105-12, doi:10.1016/j.fct.2014.07.040.

3. Könczöl M, Ebeling S, Goldenberg E, Treude F, Gminski R, Gieré R, et al. Cytotoxicity and Genotoxicity of Size-Fractionated Iron Oxide (Magnetite) in A549 Human Lung Epithelial Cells: Role of ROS, JNK, and NF-κB. Chemical Research in Toxicology. 2011;24(9):1460-75, doi:10.1021/tx200051s.

4. Manshian BB, Soenen SJ, Brown A, Hondow N, Wills J, Jenkins GJS, et al. Genotoxic capacity of Cd/Se semiconductor quantum dots with differing surface chemistries. Mutagenesis. 2015;31(1):97-106, doi:10.1093/mutage/gev061.

5. Uboldi C, Sanles Sobrido M, Bernard E, Tassistro V, Herlin-Boime N, Vrel D, et al. In Vitro Analysis of the Effects of ITER-Like Tungsten Nanoparticles: Cytotoxicity and Epigenotoxicity in BEAS-2B Cells. Nanomaterials. 2019;9(9):1233.

6. Moche H, Chevalier D, Barois N, Lorge E, Claude N, Nesslany F. Tungsten Carbide-Cobalt as a Nanoparticulate Reference Positive Control in In Vitro Genotoxicity Assays. Toxicological Sciences. 2013;137(1):125-34, doi:10.1093/toxsci/kft222.

7. Kumari M, Kumari SI, Kamal SSK, Grover P. Genotoxicity assessment of cerium oxide nanoparticles in female Wistar rats after acute oral exposure. Mutation Research/Genetic Toxicology and Environmental Mutagenesis. 2014;775-776:7-19, doi:https://doi.org/10.1016/j.mrgentox.2014.09.009.

8. Singh SP, Chinde S, Kamal SSK, Rahman MF, Mahboob M, Grover P. Genotoxic effects of chromium oxide nanoparticles and microparticles in Wistar rats after 28 days of repeated oral exposure. Environmental Science and Pollution Research. 2016;23(4):3914-24, doi:10.1007/s11356-015-5622-0.

9. Singh SP, Rahman MF, Murty US, Mahboob M, Grover P. Comparative study of genotoxicity and tissue distribution of nano and micron sized iron oxide in rats after acute oral treatment. Toxicol Appl Pharmacol. 2013;266(1):56-66, doi:10.1016/j.taap.2012.10.016.

10. Mangalampalli B, Dumala N, Grover P. Acute oral toxicity study of magnesium oxide nanoparticles and microparticles in female albino Wistar rats. Regulatory Toxicology and Pharmacology. 2017;90:170-84, doi:https://doi.org/10.1016/j.yrtph.2017.09.005.

11. Singh SP, Kumari M, Kumari SI, Rahman MF, Mahboob M, Grover P. Toxicity assessment of manganese oxide micro and nanoparticles in Wistar rats after 28 days of repeated oral exposure. Journal of Applied Toxicology. 2013;33(10):1165-79, doi:https://doi.org/10.1002/jat.2887.

12. Vales G, Rubio L, Marcos R. Genotoxic and cell-transformation effects of multi-walled carbon nanotubes (MWCNT) following in vitro sub-chronic exposures. J Hazard Mater. 2016;306:193-202, doi:10.1016/j.jhazmat.2015.12.021.

13. Di Giorgio ML, Di Bucchianico S, Ragnelli AM, Aimola P, Santucci S, Poma A. Effects of single and multi walled carbon nanotubes on macrophages: cyto and genotoxicity and electron microscopy. Mutat Res. 2011;722(1):20-31, doi:10.1016/j.mrgentox.2011.02.008.

14. Ema M, Imamura T, Suzuki H, Kobayashi N, Naya M, Nakanishi J. Evaluation of genotoxicity of multi-walled carbon nanotubes in a battery of in vitro and in vivo assays. Regul Toxicol Pharmacol. 2012;63(2):188-95, doi:10.1016/j.yrtph.2012.03.014.

15. Naya M, Kobayashi N, Mizuno K, Matsumoto K, Ema M, Nakanishi J. Evaluation of the genotoxic potential of single-wall carbon nanotubes by using a battery of in vitro and in vivo genotoxicity assays. Regul Toxicol Pharmacol. 2011;61(2):192-8, doi:10.1016/j.yrtph.2011.07.008.

16. Jacobsen NR, Møller P, Jensen KA, Vogel U, Ladefoged O, Loft S, et al. Lung inflammation and genotoxicity following pulmonary exposure to nanoparticles in ApoE-/- mice. Part Fibre Toxicol. 2009;6:2, doi:10.1186/1743-8977-6-2.

17. Poulsen SS, Jackson P, Kling K, Knudsen KB, Skaug V, Kyjovska ZO, et al. Multi-walled carbon nanotube physicochemical properties predict pulmonary inflammation and genotoxicity. Nanotoxicology. 2016;10(9):1263-75, doi:10.1080/17435390.2016.1202351.

18. Rahman L, Jacobsen NR, Aziz SA, Wu D, Williams A, Yauk CL, et al. Multi-walled carbon nanotube-induced genotoxic, inflammatory and pro-fibrotic responses in mice: Investigating the mechanisms of pulmonary carcinogenesis. Mutat Res Genet Toxicol Environ Mutagen. 2017;823:28-44, doi:10.1016/j.mrgentox.2017.08.005.
